# Equine pegiviruses cause persistent infection of bone marrow and are not associated with hepatitis

**DOI:** 10.1371/journal.ppat.1008677

**Published:** 2020-07-10

**Authors:** Joy E. Tomlinson, Raphael Wolfisberg, Ulrik Fahnøe, Himanshu Sharma, Randall W. Renshaw, Louise Nielsen, Eiko Nishiuchi, Christina Holm, Edward Dubovi, Brad R. Rosenberg, Bud C. Tennant, Jens Bukh, Amit Kapoor, Thomas J. Divers, Charles M. Rice, Gerlinde R. Van de Walle, Troels K. H. Scheel

**Affiliations:** 1 Baker Institute for Animal Health, College of Veterinary Medicine, Cornell University, Ithaca, New York, United States of America; 2 Copenhagen Hepatitis C Program (CO-HEP), Department of Infectious Diseases, Hvidovre Hospital and Department of Immunology and Microbiology, University of Copenhagen, Copenhagen, Denmark; 3 Center for Vaccines and Immunity, Research Institute at Nationwide Children’s Hospital, Columbus, Ohio, United States of America; 4 Department of Population Medicine and Diagnostic Sciences, College of Veterinary Medicine, Cornell University, Ithaca, New York, United States of America; 5 Laboratory of Virology and Infectious Disease, The Rockefeller University, New York, New York, United States of America; 6 Department of Microbiology, Icahn School of Medicine at Mount Sinai, New York, New York, United States of America; 7 Department of Clinical Sciences, College of Veterinary Medicine, Cornell University, Ithaca, New York, United States of America; University of Wisconsin, UNITED STATES

## Abstract

Pegiviruses frequently cause persistent infection (as defined by >6 months), but unlike most other *Flaviviridae* members, no apparent clinical disease. Human pegivirus (HPgV, previously GBV-C) is detectable in 1–4% of healthy individuals and another 5–13% are seropositive. Some evidence for infection of bone marrow and spleen exists. Equine pegivirus 1 (EPgV-1) is not linked to disease, whereas another pegivirus, Theiler’s disease-associated virus (TDAV), was identified in an outbreak of acute serum hepatitis (Theiler’s disease) in horses. Although no subsequent reports link TDAV to disease, any association with hepatitis has not been formally examined. Here, we characterized EPgV-1 and TDAV tropism, sequence diversity, persistence and association with liver disease in horses. Among more than 20 tissue types, we consistently detected high viral loads only in serum, bone marrow and spleen, and viral RNA replication was consistently identified in bone marrow. PBMCs and lymph nodes, but not liver, were sporadically positive. To exclude potential effects of co-infecting agents in experimental infections, we constructed full-length consensus cDNA clones; this was enabled by determination of the complete viral genomes, including a novel TDAV 3’ terminus. Clone derived RNA transcripts were used for direct intrasplenic inoculation of healthy horses. This led to productive infection detectable from week 2–3 and persisting beyond the 28 weeks of study. We did not observe any clinical signs of illness or elevation of circulating liver enzymes. The polyprotein consensus sequences did not change, suggesting that both clones were fully functional. To our knowledge, this is the first successful extrahepatic viral RNA launch and the first robust reverse genetics system for a pegivirus. In conclusion, equine pegiviruses are bone marrow tropic, cause persistent infection in horses, and are not associated with hepatitis. Based on these findings, it may be appropriate to rename the group of TDAV and related viruses as EPgV-2.

## Author summary

Transmissible hepatitis in horses (Theiler’s disease) has been known for 100 years without knowledge of causative infectious agents. Recently, two novel equine pegiviruses (EPgV) were discovered. Whereas EPgV-1 was not associated to disease, the other was identified in an outbreak of acute serum hepatitis and therefore named Theiler’s disease-associated virus (TDAV). This finding was surprising since human and monkey pegiviruses typically cause long-term infection without associated clinical disease. Whereas no subsequent reports link TDAV to disease, the original association to hepatitis has not been formally examined. Here, we studied EPgV-1 and TDAV and found that their natural history of infection in horses were remarkably similar. Examination of various tissues identified the bone marrow as the primary site of replication for both viruses with no evidence of replication in the liver. To exclude potential effects of other infectious agents, we developed molecular full-length clones for EPgV-1 and TDAV and were able to initiate infection in horses using derived synthetic viral genetic material. This demonstrated long-term infection, but no association with hepatitis. These findings call into question the connection between TDAV, liver infection, and hepatitis in horses.

## Introduction

Pegiviruses are positive-stranded RNA viruses that typically cause persistent infection (as defined by >6 months), but without apparent clinical disease [1]. As members of the *Flaviviridae* family, they are related to hepaciviruses, such as hepatitis C virus (HCV), pestiviruses, and flaviviruses. A number of species are infected by pegiviruses [2], which include human (HPgV) [3, 4], simian (SPgV) [5, 6], bat (BPgV) [7, 8], rodent (RPgV) [9–11], equine (EPgV) [12, 13] and porcine (PPgV) [14] pegivirus. In the search for human hepatitis viruses, GBV-A and -B were identified after passaging an acute phase sample from a patient (G.B.) with hepatitis in tamarins [5]. It remains unlikely that either is a human virus, and whereas GBV-B belongs to the hepaciviruses causing hepatitis, GBV-A has been classified as a simian pegivirus with no apparent disease association [1]. When a human GBV-A related virus subsequently was discovered, it was termed GBV-C (now HPgV-1) despite its lack of relation to the G.B. sample [3, 4].

HPgV-1 is detected in 1–4% of healthy blood donors, and another 5–13% are seropositive. In certain geographical regions, viremia prevalence approaches 20% [1, 15]. Recently, a divergent HPgV-2 was discovered [16, 17]. There is no evidence of clinical pathology in humans associated with the highly prevalent HPgV. Around 80% of immunocompetent individuals clear HPgV infection within 2 years of infection [18, 19], but the virus can persist for decades. Antibodies are usually not detected during infection, but appearance of anti-E2 antibodies is associated with HPgV clearance [20]. Several studies suggest bone marrow and spleen as primary target organs [21–23], with HPgV RNA also identified in lymphocyte subsets [24], but not in liver as initially suggested [3]. Cultivation of HPgV has been attempted in peripheral blood mononuclear cells (PBMCs), but replication is poor and reduced by T-cell activation [24, 25]. These studies, combined with *ex vivo* transfection of a full-length HPgV clone [26], suggest that naïve CD4+ T-cells might be permissive for HPgV [15]. Recent studies with experimental SPgV infection of macaques did not find activation of the immune system and confirmed high levels of viral RNA, specifically in bone marrow and spleen [27, 28]. Attention has been given to the putative immunomodulatory impact of HPgV co-infection in improving prognosis for human immune-deficiency virus (HIV)-positive individuals [15]. In an attempt to understand putative interactions with HIV infection, SPgV-infected macaques were co-infected with simian immune-deficiency virus (SIV). In this model, SPgV had no effect on SIV pathogenesis, at least not during acute-phase disease [28].

We first described EPgV-1 in 2013 as a naturally occurring persistent infection in horses and characterized the genome of the C35 prototype isolate [12]. Like for other pegiviruses, its genome consists of 5’ and 3’ untranslated regions (UTRs) flanking a single long open reading frame (ORF), which is cleaved into predicted structural (E1 and E2) and non-structural (X, NS2, NS3, NS4A, NS4B, NS5A and NS5B) proteins. No capsid protein is predicted, and instead a short peptide is encoded at the N-terminus. EPgV-1 RNA is found in horses world-wide at high prevalence: 6–32% in the US [12], 14% in Germany [29], 4% in the UK [30], and 1–14% in China and Brazil [31–33]. Up to 67% of horses were found to be seropositive in a UK study [30]. Most commercial horse sera for cell culture and serum-derived equine biological products are EPgV positive [29]. Another pegivirus with only ~40% amino acid sequence identity was identified in an outbreak of acute hepatitis in horses (a.k.a. Theiler’s disease) [34] following administration of equine-produced antitoxin plasma, and provisionally named Theiler’s disease-associated virus (TDAV) [13]. This plasma-transferred virus showed persistent infection in ~30% of infected horses. Here, we will use “TDAV” in reference to this specific isolate, and “EPgV-2” in reference to this group of pegiviruses. Prevalence of EPgV-2 RNA is generally low, e.g. 1.6% in Brazil [35], and not present in samples collected in other studies [29–32]. EPgV-2 RNA, however, has been identified in commercial serum pools [29].

Since pegiviruses generally are not associated with clinical disease, it was a surprise when TDAV was found in an outbreak of acute serum hepatitis [13]. No additional links between EPgV-2 and clinical cases of equine liver disease have been reported; instead a recently discovered parvovirus (EqPV-H) appears responsible for a number of Theiler’s disease cases in horses [36–41]. So far, however, the possible link between equine pegiviruses and hepatitis has not been formally examined. With the aim to investigate the natural history of EPgV infection and any further relation between TDAV/EPgV-2 and liver disease in horses, we here compared viral load, sequence diversity, persistence, tissue tropism, course of infection, and association to liver disease for EPgV-1 and -2 in horses.

## Results

### Equine pegivirus viral load, sequence diversity and persistence

**Fig 1:**
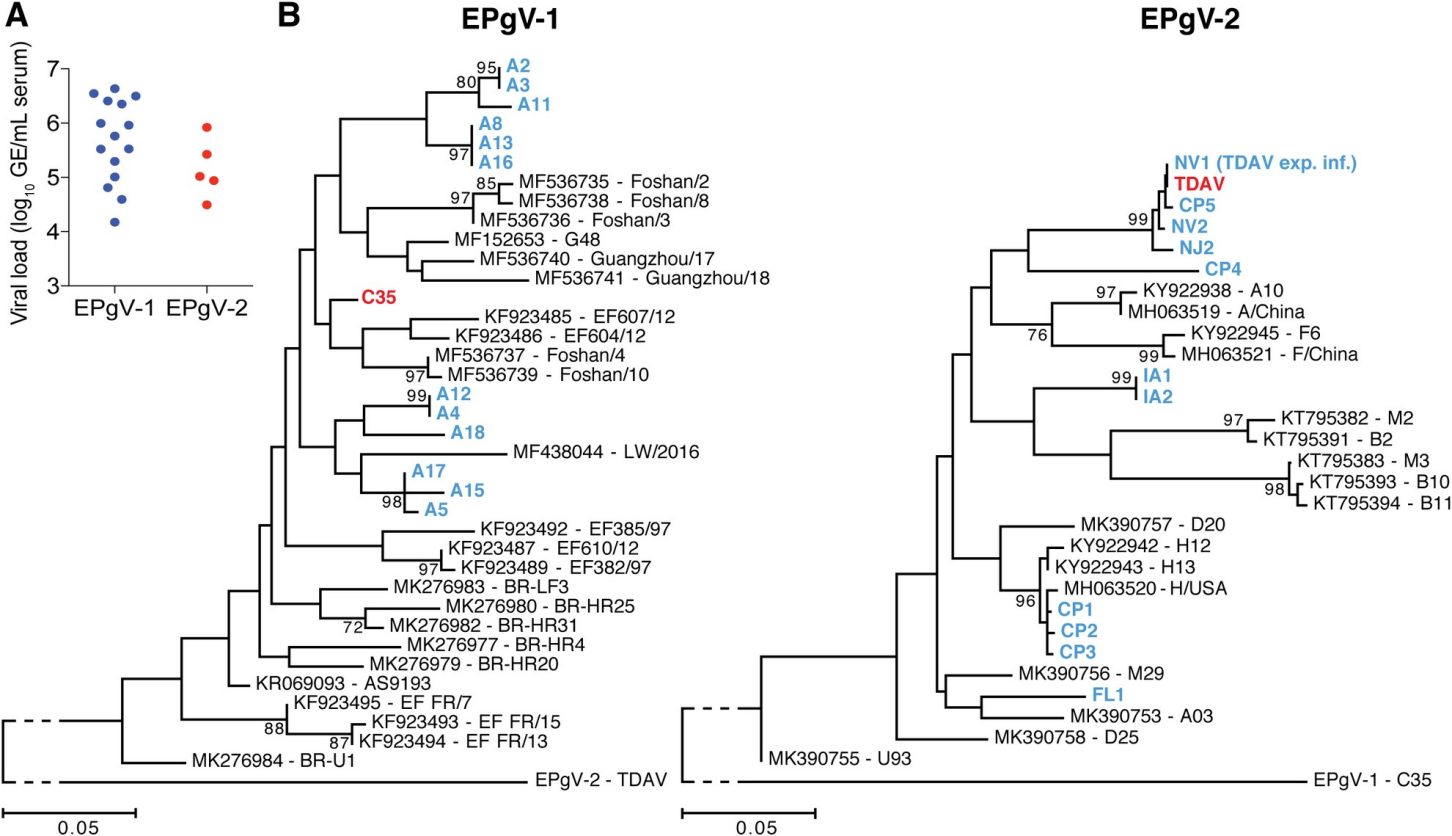
EPgV viral load and diversity. **(A)** EPgV-1 and EPgV-2 RNA genome equivalents (GE) per mL horse serum were determined for individual animals. **(B)** Phylogeny of partial NS3 nucleotide sequences of EPgV-1 (nt 4555–4768; numbering according to C35 genome MT276211) and EPgV-2 (nt 3712–4128; numbering according to TDAV genome MT276199) inferred using the Maximum Likelihood method in MEGA7. The evolutionary distances are in the units of the number of nucleotide substitutions per site. Bootstrap values of at least 70 are shown. Prototype strains are highlighted in red. Isolates sequenced in this study are in blue. CP: commercial product; all others are from individual animals. Published sequences are in black with GenBank entry and strain name indicated. When several sequences from the same publication were identical only one representative sequence was included in the tree.

We initially determined viral load in serum samples from individual infected horses identified during investigations at specific farms ([12, 13] and current study) at unknown duration of infection. Viral loads of both pegiviruses were similar, with EPgV-1 ranging from ~1x10^4^ to 4x10^6^ genome equivalents (GE)/mL, and EPgV-2 ranging from ~3x10^4^ to 1x10^6^ GE/mL (**Fig 1A**). This is lower than the 10^6^−10^7^ GE/mL typically observed for HPgV [42] and SPgV [27].

To further our understanding of EPgV diversity, we determined partial NS3 sequences from twelve EPgV-1 and eleven EPgV-2 samples, including samples from Fig 1A and five commercial products. Phylogenetic analysis comparing these isolates and sequences reported in the literature [29–31, 33, 35, 43–45], revealed a similar diversity for EPgV-1 and -2 isolates (**Fig 1B**). Since only a few EPgV-2 near full-length sequences have been reported [13, 45], we further determined the almost complete EPgV-2 genome sequences of two chronic carriers. These IA1 and IA2 isolates were nearly identical (see also NS3 phylogeny in Fig 1B), and diverged by 8.5% (nt) and 1.6% (aa) from the original TDAV sequence [13].

To further study infection duration and seroconversion, we retrospectively examined samples from two farms with EPgV-2 infections. On the farm where the two IA samples originated, 13 of 83 horses were EPgV-2 infected at the time of sampling. Retrospective analysis of banked samples from those 13 horses revealed that five (38%) were recently infected within the past 4 months, while eight (62%) had been infected at least for the 4–12 months sampled, showing that long duration of infection is common. Further, banked samples from nine horses that were inadvertently infected by administration of TDAV contaminated antitoxin [13], showed that four (44%) were still viremic two years after infection. We also analyzed the presence of anti-NS3 antibodies in these nine samples using the luciferase immunoprecipitation system (LIPS) assay, and found only one horse seropositive two years after infection. Interestingly, this sole seropositive horse cleared its EPgV-2 viremia within the following 4 months. Finally, among four TDAV experimentally infected ponies [13], we found that only one seroconverted during the 10-week study period. These observations conform with previous findings of persistent pegivirus infection [1], and do not suggest a correlation between the presence of NS3 specific antibodies and current or previous EPgV-2 infection. It remains to be determined whether E2-specific antibodies would be a better marker, as is the case for HPgV/GBV-C [1].

### Equine pegiviruses replicate in the bone marrow

**Fig 2:**
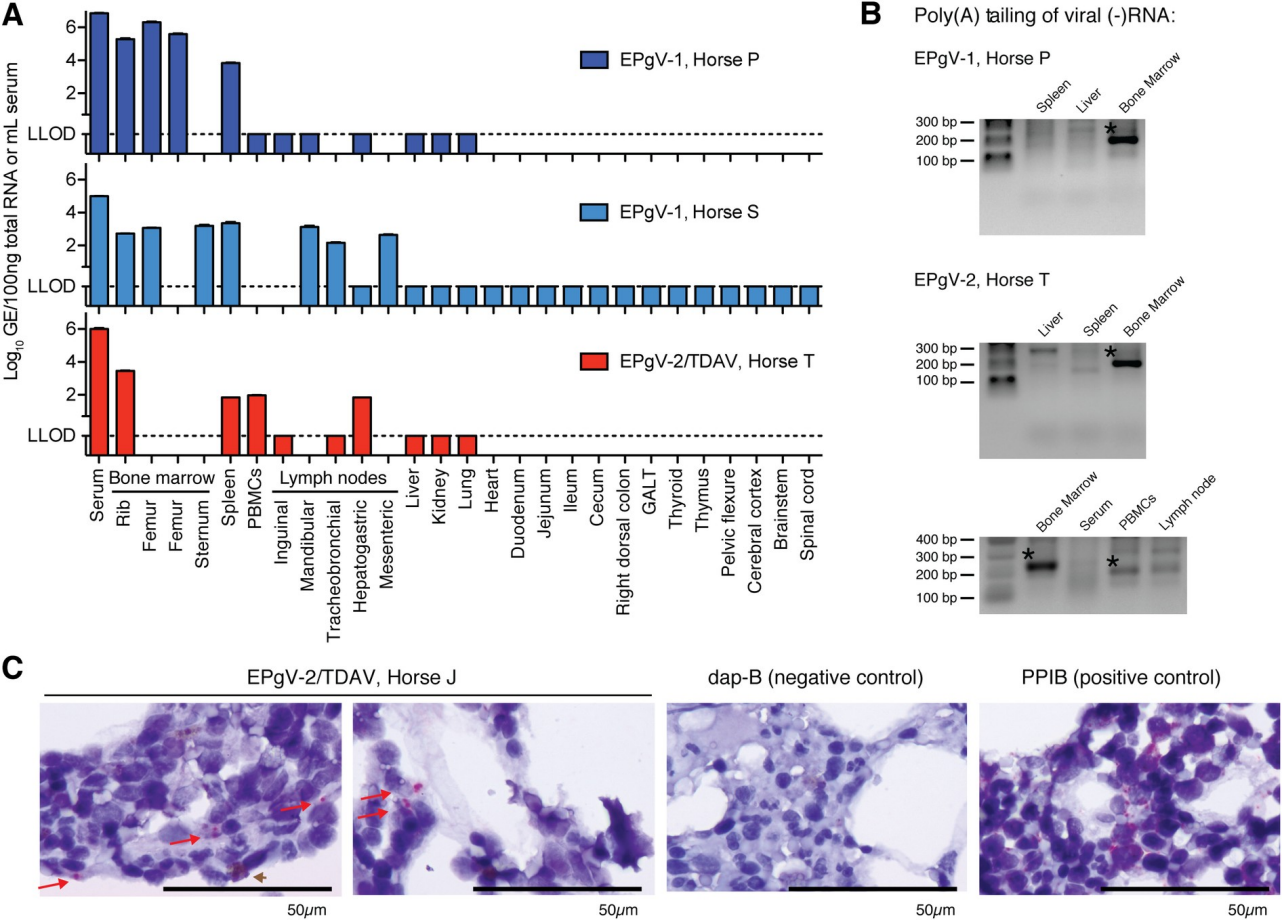
EPgV tissue tropism and site of replication. **(A)** Viral RNA levels in serum and tissues. EPgV-1 sampling was done on naturally infected horses positive for at least 8 (Horse P) and 1 (Horse S) months. EPgV-2 sampling was done 29 days post experimental infection with TDAV (except PBMCs on day 28; Horse T). LLOD: lower limit of detection. **(B)** Negative-strand specific tailing-based RT-PCR performed on bone marrow, spleen and liver for EPgV-1 (Horse P) and bone marrow, spleen, liver, PBMCs and tracheobronchial lymph node for EPgV-2 (Horse T). Expected size of PCR products are 200 and 220 base pairs for EPgV-1 and EPgV-2, respectively. Bands with confirmed viral sequence are indicated with an asterisk. **(C)** *In situ* hybridization (ISH) on bone marrow core biopsies for TDAV (Horse J), Dap-B (negative control) and PPIB (positive control) on 1000x magnification images. Images are from sampling on week 5 post inoculation (see lower panel of Fig 4A). Positive pink foci are indicated with red arrows. Note that a reddish-brown pigment (brown arrowhead) in some instances is difficult to distinguish from positive probe labelling. Scale bars represent 50μm.

The tissue tropism of equine pegiviruses remains poorly characterized. We therefore quantified viral RNA across a range of tissues from three infected horses. For EPgV-1, samples from two naturally infected horses were evaluated. Both horses were negative for EPgV-2, equine hepacivirus/non-primate hepacivirus (EqHV/NPHV) and EqPV-H nucleic acids, but were EqHV seropositive. One horse was a 13-year-old Thoroughbred gelding (Horse P), the other a 16-year-old Thoroughbred mare (Horse S), which at the time of euthanasia had been EPgV-1 positive for at least 8 and 1 months, respectively. For EPgV-2, samples from a 19-year-old quarter horse mare (Horse T) were evaluated. This horse was negative for EPgV-1, EqHV and EqPV-H nucleic acids, but EqHV seropositive, and had been experimentally inoculated with 8.9x10^7^ GE TDAV positive donor serum [13]. Horse T had normal blood count and serum liver biomarkers prior to inoculation (see methods for details) and was TDAV positive at day 7, the first day of sampling post inoculation. On day 29, the horse was euthanized and tissue samples were collected. Only serum, bone marrow and spleen samples were consistently positive for viral RNA for both viruses. Lower signal was observed in some cases for PBMCs and lymph nodes (**Fig 2A**). To unambiguously identify sites of viral replication, positive tissue compartments from Horse P (EPgV-1) and Horse T (EPgV-2), as well as liver, were further tested for the presence of negative-strand (-)RNA, a hallmark of replication for positive-strand RNA viruses. Using a 3’ end tailing assay, we consistently identified (-)RNA in bone marrow, and not in spleen or liver (**Fig 2B**). Sequencing of PCR products confirmed amplification of (-)RNA. PBMC and lymph node samples from Horse T gave weak bands, of which viral RNA could be confirmed only for PBMCs.

To visualize pegivirus infection of the bone marrow, we probed core biopsies using RNAScope *in situ* hybridization (ISH). For EPgV-2, we observed hybridization in a minority of cells (**Fig 2C**). Unambiguous detection of hybridization could not be established for EPgV-1 samples, due to lower probe signal intensity and background pigment (likely hemosiderin in macrophages) of a similar color (**S1 Fig**). Given that double-stranded RNA (dsRNA) is a typical replication intermediate for positive-stranded RNA viruses, immunohistochemistry (IHC) with anti-dsRNA antibody (J2) was performed. This convincingly confirmed punctate signal for the EPgV-2, but not for the EPgV-1 samples (**S1 Fig**). Due to lack of IHC reagents optimized for specific cell type identification in formalin fixed equine bone marrow tissue, we were not able to identify the specific cells positive for EPgV-2 ISH/IHC signal. However, the morphology of typical positive cells was consistent with that of early myeloid, macrophage, or spindle cell lineages, and not with that of cells with segmented nuclei (e.g. granulocytes).

From these tropism data, we conclude that the primary tissue tropism of equine pegiviruses is the bone marrow. Due to inconsistent detection of viral (+)/(-)RNA in spleen, PBMCs and lymph nodes, it appears that EPgV infected immune cells, potentially migrating from the bone marrow, can be present in other compartments. These findings align with studies on SPgV, where bone marrow was also identified as the primary site of replication [27]. Moreover, our data suggest that TDAV does not replicate in the liver, which is in accordance with recent studies identifying EqPV-H, and not TDAV, in a series of Theiler’s disease cases in horses [36, 37].

### Determination of EPgV complete genomic ends

**Fig 3:**
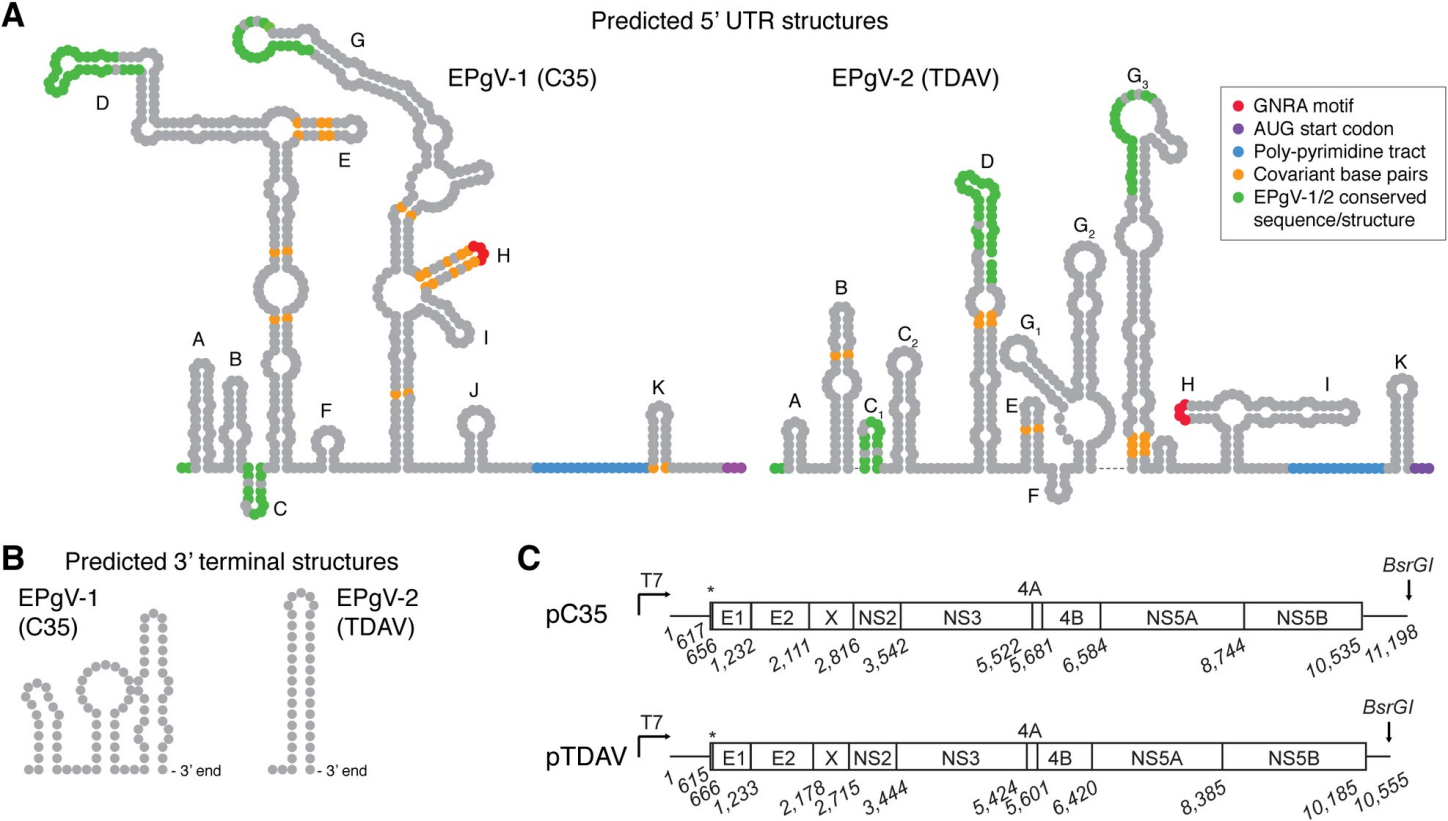
Structures of EPgV RNA ends and consensus clone genomes. **(A)** Prediction of RNA secondary structures of the C35 and TDAV 5’ UTRs were performed using MFOLD [59] and guided by covariant base-pairs from alignment of isolates. Stem-loops are labelled according to previous EPgV-1 structure prediction [12]; corresponding labelling of TDAV stem-loops is attempted. Only little direct similarity is evident between 5’ UTR structures of EPgV-1 and -2; this is indicated in green. Other features are colored as indicated. **(B)** Predicted 3’ UTR terminal stem-loops. **(C)** Schematic outline of the pC35 and pTDAV molecular consensus clones drawn to scale with nucleotide numbering of junctions predicted from alignment to other pegiviruses and using SignalP 5.0 [60]. Asterisks indicate the N-terminal peptide of the polyprotein. High resolution structures and alignments of UTRs are shown in S2 and S3 Figs.

We previously determined the complete EPgV-1 genomic ends and predicted their structure [12]. Here, sequencing of tailed 3’ ends of positive and negative RNA strands confirmed the previously determined ends, except for an additional nucleotide in the polypyrimidine tract of the internal ribosome entry site (IRES). To validate the 5’ end of EPgV-2, we further performed 5’ rapid amplification of cDNA ends (RACE) of serum derived TDAV (+)RNA and 3’ end tailing of bone marrow-derived (-)RNA. These two independent methods agreed on the 5’ end, which was similar to the published TDAV sequence [13], except for the absence of nts previously reported at positions 1, 25 and 151 of sequence KC145265. When the predicted structure for the C35 isolate was compared to our previously published structure based on EPgV-1 alignment [12], some differences were found on the D and G loops. Structure prediction, aided by covariant base-pairs between isolates, suggested an EPgV-2 IRES typical for pegiviruses, but with surprisingly limited identity to the EPgV-1 structure (**Fig 3A and S2 Fig**).

Lack of a characteristic stable stem-loop structure at the 3’ terminus of the published TDAV sequence [13] suggested that the 3’ UTR might be incomplete. Using tailing of the 3’ end of serum-derived (+)RNA, we identified an additional 80 nts downstream of the previously determined sequence. This sequence was predicted to fold into a highly stable 3’ terminal stem-loop structure (**Fig 3B, S2 Fig** and **S3 Fig**), reminiscent of those found for most non-polyadenylated RNA viruses, including members of the *Flaviviridae*. Sequencing of the IA2 isolate revealed high 3’ UTR sequence conservation to the TDAV isolate, including two poly(C) tracts, but no presence of repeat sequence elements (RSE) as observed for EPgV-1 [12]. Some nucleotide variation was observed between the two EPgV-2 isolates, in particular in the first part of the 3’ UTR, although less than for EPgV-1 (**S3 Fig**).

### Construction of EPgV full-length consensus clones and lack of replication *in vitro*

Having identified the complete EPgV genome ends, we set out to construct full-length consensus clones. For EPgV-1, we used the C35 isolate [12], and constructed a full-length molecular clone of 11,198 nts encoding a polyprotein of 3,305 amino acids with a 5’ UTR of 616 nts and a 3’ UTR of 664 nts. For EPgV-2, we used the TDAV isolate [13], which has a total length of 10,555 nts encoding a polyprotein of 3,189 amino acids with a 5’ UTR of 614 nts and a 3’ UTR of 371 nts. Both clones were flanked by a T7 promoter for *in vitro* transcription and a *BsrGI* restriction site for linearization (**Fig 3C**).

To assess replication *in vitro*, we transfected *in vitro* transcribed C35 RNA into human hepatoma (Huh-7.5), bovine kidney (MDBK), baby hamster kidney (BHK-J) and African green monkey kidney (Vero) cells, as well as an equine fibroblast cell line (E. Derm). No viral replication was observed, as assessed by RT-qPCR of cells transfected with C35 or a polymerase defective C35-GNN mutant (**S4 Fig**). Given the previous assumption of TDAV liver tropism, we further inoculated primary equine fetal liver cells (EFLCs) [46] with TDAV-positive serum. As expected, based on the absence of EPgV in the liver samples from our tropism study, no TDAV replication was detected by RT-qPCR. As we did not have access to equine bone marrow-derived primary or immortalized cell lines, investigation of EPgV replication *in vitro* was not pursued further.

### EPgV full-length consensus clones are infectious in horses

**Fig 4:**
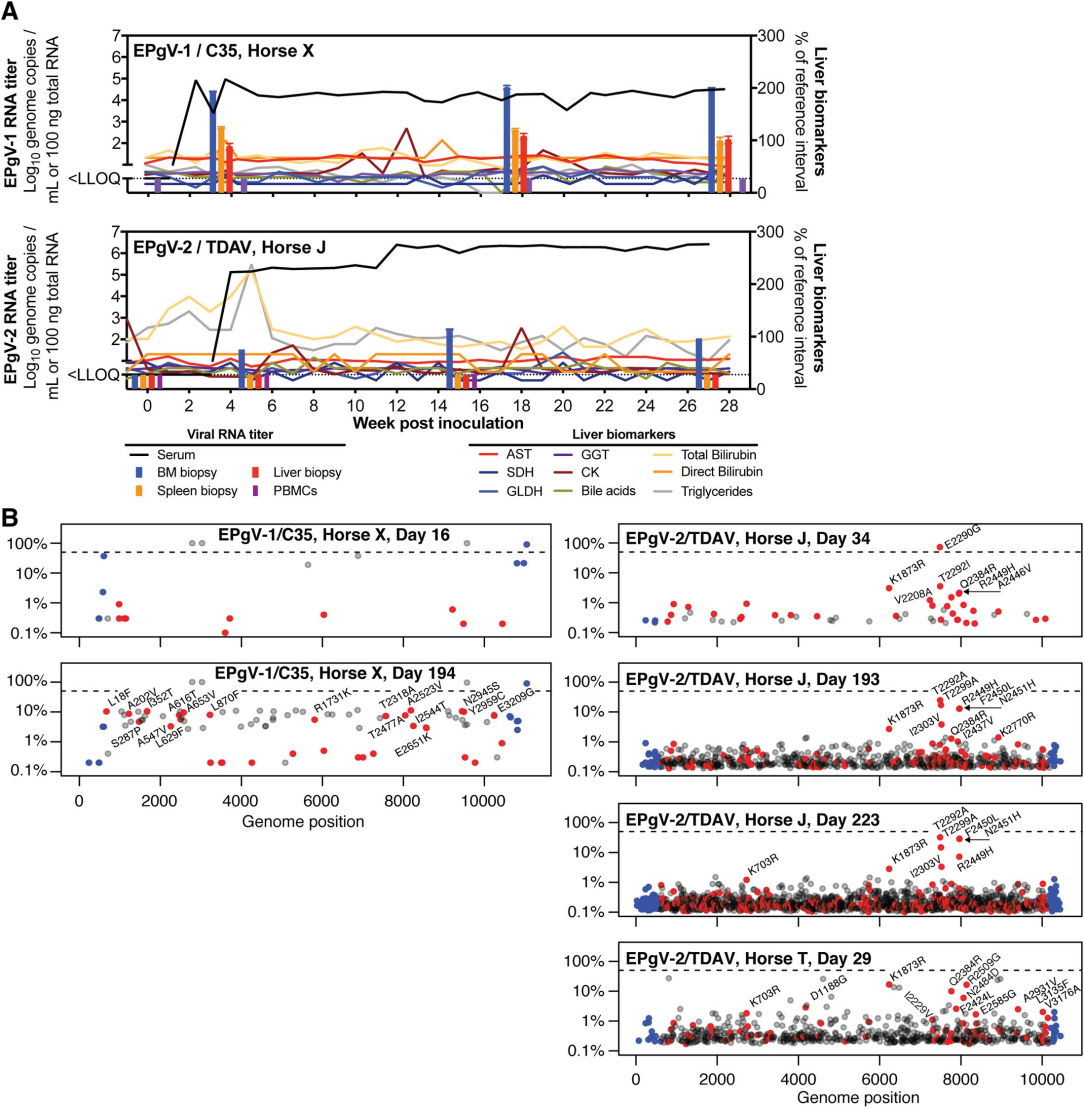
Experimental EPgV infection in horses by inoculation of genomic RNA. **(A)** Course of infection after intrasplenic inoculation of *in vitro* transcribed C35 (Horse X) or TDAV (Horse J) RNA. Liver biomarkers are plotted as percentage of reference interval maximum (AST, 222–489 U/L; SDH, 0–6 U/L; GLDH, 2–10 U/L; GGT, 8–33 U/L; bilirubin, 0.1–0.3 mg/dL (direct); creatine kinase, 171–567 U/L). RNA levels in tissue biopsy samples are shown as bars. LLOQ: lower limit of quantification. **(B)** Viral genome diversity as characterized by deep sequencing over time for the C35 (Horse X, left) and TDAV (Horse J, right) RNA inoculated horses and a TDAV infected horse (Horse T) for comparison (lower right). The frequency of deviations from consensus are plotted on a logarithmic scale with non-synonymous mutations in red, synonymous in grey and mutations in the UTRs in blue. Non-synonymous mutations with a frequency >1% are labelled. The dashed line indicates 50% (consensus change).

The availability of full-length consensus clones provides a unique opportunity to study whether RNA transcripts can initiate infection *in vivo*, and to study the course of equine pegivirus infection unaffected by co-administration of co-circulating pathogens. Successful infection by inoculation of clone-derived RNA into the liver was previously demonstrated for hepatotropic viruses, including HCV in chimpanzees [47, 48] and EqHV in horses [46]. Given the tropism, organ accessibility and animal safety, we opted for intrasplenic rather than bone marrow RNA inoculation. First, we identified a horse eligible for TDAV infection that was negative for EPgV-1/2, EqHV and EqPV-H nucleic acids and EPgV-2 NS3 antibodies (Horse J). Laparoscopic guided TDAV RNA inoculation into five sites of the spleen established productive infection (**Fig 4A**). Although no viremia was observed during the first three weeks, viral titers of ~10^5^ GE/mL serum were quantified from week 4 further increasing to ~10^6^ GE/mL from week 12 onwards. TDAV RNA was identified in bone marrow biopsies during acute and chronic phase viremia at weeks 5, 15 and 27, but not in spleen, liver or PBMCs. The horse was followed for 28 weeks and remained infected throughout the study period (**Fig 4A**).

Due to the lack of an EPgV-1-specific serology assay, we were unable to pre-screen for previous EPgV-1 exposure to identify a horse eligible for C35 infection. Initial attempts of intrasplenic RNA inoculation into a 4-year old mare and an 8-year old gelding that were both negative for EPgV-1/2, EqHV and EqPV-H nucleic acids were unsuccessful. In case the spleen did not contain permissive cells, inoculation into the bone marrow of the gelding was also attempted, but unsuccessful. However, since intravenous challenge with EPgV-1 infectious serum also did not lead to infection in that horse, we ascribed the lack of successful infection to pre-existing immunity. In contrast, C35 RNA inoculation into five splenic sites of a 2-year-old mare (Horse X), which was negative for EPgV-1/2, EqHV and EqPV-H nucleic acids, did result in productive infection (**Fig 4A**). Viral titers between 10^4^−10^5^ GE/mL serum were observed from week 2 onwards. Bone marrow biopsies during acute and chronic phase viremia at weeks 4, 18 and 28 were positive for viral RNA, with lower titers observed in spleen and liver. No viral RNA could be detected in PBMCs. This horse was also followed for 28 weeks and remained infected throughout the study period.

To study evolution of the viral genomes, we performed deep sequencing of the entire ORF at early acute and late (~6 months follow-up) time points (**Fig 4B and S1 Data**). We noticed an increasing number of low abundance variants over time, although less so for C35 (potentially explained by lower titers and thus, lower sampling depth). For C35, three non-coding changes to the input consensus were observed already from day 16. In addition, a poly(C) tract in the 3’ UTR expanded from 7 to 8 Cs. No protein level consensus changes were observed, but a sub-population with several amino acid differences constituted ~10% on day 194. For TDAV, no changes to the input consensus were observed, except for a temporary E2290G mutation in NS5A dominating on day 34. Non-synonymous changes with prevalence >1% throughout the course of infection were observed for residue 1873 in NS4B and in the C-terminus of NS5A (residue 2290–2451). Interestingly, similar variation for residues 1873 and 2384 was observed for the experimentally infected horse used for tissue tropism studies (Horse T, **Fig 4B and S1 Data**). For both Horse T (day 29) and a horse infected with the original TDAV containing antitoxin (NV1 of Fig 1B) [13], no changes to the consensus sequence were found. In addition, the complete genomic C35 and TDAV ends were sequenced for the acute phase samples, and no consensus changes were observed. Thus, the consensus clones were infectious and the viral sequence displayed high stability.

### EPgV infections are clinically silent

No clinical illness and no elevated liver markers were observed for the two successfully transfected horses **(Fig 4A)**. In addition, acute inflammatory markers including serum amyloid A and iron indices (serum iron concentration, total iron binding capacity, and percent saturation of transferrin) were measured weekly for 8 weeks after transfection and remained within normal range. A brief increase in total and indirect bilirubin for the TDAV-transfected horse could be explained by an initial inappetence while housed in isolation.

**Fig 5:**
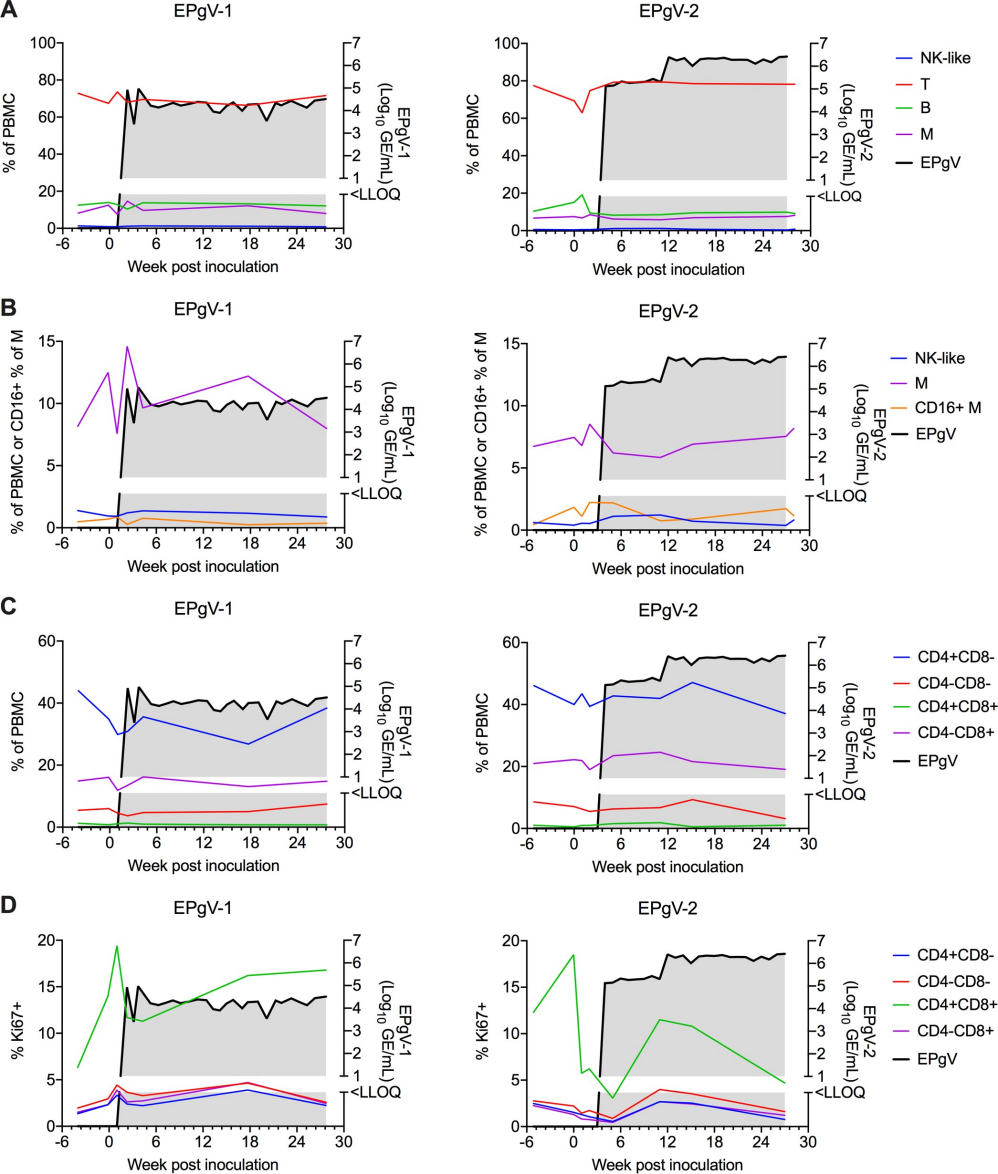
Phenotyping of PBMCs during EPgV infection. **(A)** Major cell populations over the course of infection after intrasplenic inoculation of C35 (EPgV-1) or TDAV (EPgV-2) RNA. PBMCs were isolated before inoculation, weeks 0, 1, and 2 after inoculation, during acute viremia (week 4–5), during chronic viremia (week 15–17), and at end of study (week 27–28). NK-like cells were defined as CD3^+^CD16^+^. No CD3^-^CD14^-^CD16^+^ cells were identified. **(B)** Alternatively activated monocytes, defined as CD14^+^CD16^+^, over the course of infection. Smaller populations (M, NK-like) are also shown in greater detail here. **(C)** CD3^+^ cells over the course of infection, subdivided by CD4 and CD8 expression. **(D)** The proportion of proliferating (Ki67^+^) CD3^+^ cells over the course of infection. A, B determined from flow panel M (**S5 Fig**). C, D determined from panel T (**S6 Fig**). NK-like: natural killer-like T: T-cells; B: B-cells; M: monocytes.

For both transfected horses, PBMCs were immunophenotyped by flow cytometry to identify and quantify major cell populations, including NK-like cells, T-cells, B-cells, and monocytes (**Fig 5A**). In addition, alternatively activated monocytes were identified by CD16^+^ (**Fig 5B**), and T-cells were subdivided based on CD4 and CD8 expression (**Fig 5C**). The proportion of proliferative (Ki67^+^) cells in each of the T-cell populations was measured as well (**Fig 5D**). Overall, we did not find any clear differences or trends in any of these immune cell populations over the course of infection, although smaller changes might be identified with larger case numbers. For the TDAV-transfected horse, we further analyzed the presence of anti-NS3 antibodies by LIPS, but did not observe seroconversion within the duration of the study.

In conclusion, EPgV-1 and -2 replicate in the bone marrow causing persistent infection in horses, without clinical illness or liver abnormalities.

## Discussion

Pegiviruses, including the widespread EPgV-1, are generally not considered to be associated with disease [33, 49]. Therefore, it was a surprise when another equine pegivirus, TDAV, was found in an outbreak of acute serum hepatitis in horses [13]. To evaluate the modern version of Koch’s postulates, we constructed infectious full-length molecular clones of both equine pegiviruses, EPgV-1/C35 and EPgV-2/TDAV, to study their natural history using an RNA inoculum devoid of any other potentially co-circulating infectious agents. Inoculation in two horses demonstrated a similar course of infection for EPgV-1 and -2, with persistent infection of the bone marrow, an intermediate viral load around 10^5^ GE/mL, no overt changes to immune cell populations, and importantly, no indication of damage or infection of the liver. Given that selection of viral mutants, which potentially could evade adaptive immunity, was not observed, we did not perform functional T-cell experiments. It further remains to be established whether the overall higher viral load in tissues, despite a lower viremia, for C35 compared to TDAV is a general characteristic of EPgV-1 vs. -2 infections. Since EPgV-2 infections have also not been identified in cases of equine serum hepatitis subsequent to the initial outbreak, these data further substantiates the lack of association between TDAV and liver disease. Rather, the novel equine parvovirus, EqPV-H, was identified in many investigated cases of acute serum hepatitis, as well as other cases of acute liver disease in horses [36–39]. Interestingly, EqPV-H was retrospectively also identified in the original EPgV-2/TDAV-contaminated inoculum [36]. In addition, the HCV-related hepatotropic EqHV/NPHV does cause mild hepatitis during the acute phase in horses [46], and might also be responsible for some cases of severe liver disease in persistently infected animals [50]. It appears, however, that only a minority of infections progress to persistence [2, 49, 51].

Collectively, these findings are highly indicative that EPgVs are not associated with liver disease, but are bone marrow tropic and non-pathogenic; and thus, similar to pegiviruses of other species. We therefore propose to define equine pegiviruses as EPgV-1, to include the C35 and related isolates, and EPgV-2, to include the original TDAV and related isolates. EPgV-1 would be assigned to the proposed species “Pegivirus E” with type isolate C35 (KC410872 or MT276211), and EPgV-2 would be assigned to “Pegivirus D” with type isolate TDAV (KC145265 or MT276199) [52]. Given that HPgV originally was thought to cause hepatitis and was even named hepatitis G virus (HGV), the history of detaching a pegivirus from an apparently incorrect association with liver disease is repeating itself. Nonetheless, pegiviruses should remain of high interest given their unique ability to persistently infect their hosts without eliciting a strong enough immune response to be cleared by the immune system.

Given the absence of disease association, the tissue tropism of pegiviruses has remained understudied. Due to limited sample availability, only few studies have explored HPgV tropism in depth and although not unanimous, bone marrow and spleen were suggested primary target organs [21–23]. Other studies focused on the more easily accessible lymphocyte subsets and, based on this work, suggested e.g. naïve CD4+ lymphocytes as a potential virus reservoir [24, 25]. Since animal models better enable tropism studies, evaluation of SPgV (+)RNA levels in a large number of macaque tissues more convincingly identified bone marrow and spleen as target organs with viral RNA also present in PBMCs [27]. Interestingly, viremia remained unperturbed in most animals after splenectomy, suggesting bone marrow to be the most important tissue for SPgV replication. Here, we consistently identify high EPgV RNA titers in bone marrow, lower titers in spleen, and occasionally RNA positive PBMC and lymph node samples. Negative strand RNA, a hallmark of viral replication, was consistently identified in bone marrow but not in spleen or liver. PBMCs from Horse T (EPgV-2) were weakly positive for viral (+) and (-)RNA, whereas other PBMC samples from Horse P (EPgV-1) and the two transfected horses were negative. Thus, bone marrow is the primary site also of equine pegivirus replication. It appears, however, that lower numbers of permissive cells patrol other compartments, such as the spleen, lymph nodes, and blood, at least transiently. This would also align with tissue distribution of hematopoietic cell lineages originating from bone marrow and the fact that genomic RNA inoculation into the spleen led to productive infection. Therefore, it will be of particular interest in future studies to identify the specific cell types permissive for EPgV. Recent improvements in methodology, such as single-cell RNA sequencing, could aid such pursuit.

Our characterization of natural EPgV infections determined a viral load in the range of 10^4^ to 4x10^6^ GE/mL, which is not atypical for pegiviruses. Due to an initially low number of identified EPgV-2 sequences, the geographical distribution and diversity of this virus was not well understood. The expanded sequence collection and phylogenetic analysis presented here, however, suggest a similar diversity for the two EPgVs despite great differences in prevalence. Additionally, we present the near full-length sequences of an additional three EPgV-2 isolates (IA1, IA2 and NV1), which should be helpful for further understanding of this virus. Importantly, we also found that the previously published TDAV sequence was incomplete and identified additional 80 nts at its 3’ end. Structural predictions suggested that the 5’ UTRs of EPgV-1 and -2 both contain elements common for the type of IRES used by these viruses, consisting of a number of longer or shorter stem-loops, a GNRA motif, and a poly-pyrimidine tract separated from the start codon by a short stem-loop. Although structure predictions revealed three common loops, the remaining 5’ UTR structures shared surprisingly little similarity between the two viruses. The 3’ UTRs of both viruses contain short poly(C) tracts and terminate in highly paired stem-loop structures, similar to what is seen for HPgV and SPgV [27, 53]. In the 3’ UTR of EPgV-1, we previously identified a number of RSE predicted to fold into similar loop structures ([12] and **S3 Fig**), possibly interacting with RNA-binding proteins. For the 3’ UTR of EPgV-2, little direct similarity to EPgV-1 is seen and no RSE were identified. Additional EPgV 3’ UTR sequences from diverse isolates could help identify further structural elements based on co-variation.

The ability to initiate viral infection from a molecular clone not only fulfills the modern version of Koch’s postulates, but also allows verification of the complete, functional genome. Successful intrahepatic inoculation was previously used for HAV in marmosets [54], HCV in chimpanzees [47, 48], GBV-B in tamarins [55], EqHV/NPHV in horses [46] and RHV in rats [56]. To our knowledge, the intrasplenic launch of the EPgV infectious clones C35 and TDAV described here is the first successful extrahepatic viral RNA inoculation. It further represents the first robust reverse genetics system for a pegivirus. Although viral RNA could be detected in PBMCs *ex vivo* after transfection with a HPgV/GBV-C clone, titers were low and the system has not become widely used [26]. Given the identified bone marrow tropism of EPgVs, it was unsurprising that attempts to infect cell lines of different origins was unsuccessful. Further efforts to identify the most permissive cell types *in vivo* should mediate establishment of *in vitro* culture systems for pegiviruses. In addition to confirming the completeness and functionality of the determined C35 and TDAV sequences in our present study, these EPgV clones thus will be useful for reverse genetic studies both *in vitro and in vivo*.

In conclusion, this study found no association between equine TDAV infection and liver tropism or disease. Rather, bone marrow appears to be the most important compartment for replication of both EPgV-1 and EPgV-2. Infection is not associated with any overt changes in peripheral immune cell populations and can progress to persistence. A similar natural history was found for both EPgVs, none of which are associated with any clinical illness or liver abnormalities. The results from this study therefore contribute to the growing list of pegiviruses with limited pathogenicity identified in different species.

## Materials and methods

### Ethics statement

All animal husbandry and sampling adhered to Cornell University IACUC protocols (permit 2014–0024). Ultrasound-guided percutaneous biopsies of tissues, as indicated, were taken from selected horses using standard procedures at the College of Veterinary Medicine, Cornell University. Samples for viral load and phylogeny studies were from individual U.S. horses screened for presence of various pathogens or from commercial horse sera. Three horses were euthanized for a tropism study and tissues were collected from major organs without perfusion. Aliquots were snap frozen and stored at −80°C until further analysis.

### Isolation, quantification and sequencing of viral RNA

Horses were screened for EPgV-1/-2, EqHV/NPHV and EqPV-H nucleic acids prior to inclusion, as previously described [37]. For quantification of EPgVs, RNA was purified from serum or plasma using High Pure Viral Nucleic Acid Kit (Roche), QIAamp Viral RNA mini kit (Qiagen) or TRIzol (Life Technologies) and from tissues after disruption in MagNA lyser Green Beads vials using a MagNA lyser instrument (Roche). Viral load was quantified by one-step reverse transcription quantitative PCR (RT-qPCR) using TaqMan Fast Virus 1-Step Master Mix (Applied Bioscience) on a LightCycler (Roche) with the following cycling parameters: 50°C for 30 min, 95°C for 5 min followed by 40 cycles of 95°C for 15 s, 56°C for 30 s and 60°C for 45 s. Primers were TS-O-00373, -374 and probe -375 for EPgV-1 and TS-O-00130, -131 and probe -132 for EPgV-2. All primer sequences are listed in S1 Table. Standard curves were generated from *in vitro* transcribed RNAs from plasmids encoding partial C35 or TDAV sequences. Following *in vitro* transcription, template DNA was removed by extensive DNase treatment and purified RNA was quantified and diluted to concentrations ranging from 10^8^−10^1^ GE/μL.

For sequence determination of partial EPgV-1 NS3, RNA was reverse transcribed using SuperScript III (Thermo Fisher) and random nonamers (Sigma-Aldrich). PCR was done using AmpliTaq Gold (Thermo Fisher) and the primer set RU-O-19037 and -038 at 95°C for 8 min, 10 cycles of 95°C for 40 s, 67°C (decreasing by 0.5°C per cycle) for 45 s and 72°C for 30 s, followed by 30 cycles of 95°C for 30 s, 56°C for 40 s and 72°C for 30 s. For EPgV-2, Superscript III One step RT-PCR with Platinum Taq (ThermoFisher) was used with primers TDAV-236F and TDAV-692R at 48°C for 30 min, 95°C for 5 min, and 30 cycles of 95°C for 30 s, 59°C for 30 s and 72°C for 60 s.

To determine the complete viral 5’ ends, we employed 5’ RACE (Thermo Fisher) on serum derived viral RNA. The primers TS-O-01214 or -311 and SuperScript III were used for cDNA synthesis from EPgV-1 or EPgV-2 RNA, respectively, in a continuous gradient from 50°C to 55°C for 30 min. Independent TdT tailing reactions with two different types of dNTPs were performed for each virus. Primers for first-round PCR amplification were TS-O-00178 or AAP with TS-O-00653 for EPgV-1 dATP- or dCTP-tailing, respectively and AAP or TS-O-00525 with TS-O-00131 for EPgV-2 dCTP- or dGTP-tailing, respectively. Primer pairs for the second-round PCR were AUAP and TS-O-00654 for amplification of EPgV-1 and AUAP with TS-O-00312 for EPgV-2. PCR products were generated using Q5 Hot Start High-Fidelity 2x Master Mix (NEB) with cycling parameters 98°C for 30 s followed by 40 cycles of 98°C for 30 s, 52°C for 10 s, and 72°C for 1 min plus a final extension at 72°C for 5 min. To determine the complete 3’ ends, viral RNA was tailed with homopolymers of GTP, ATP or UTP, using Yeast Poly(A) Polymerase (USB Affymetrix), and cDNA was synthesized using SuperScript III and primers RU-O-18176 (G-tailing), TS-O-00178 (A-tailing) or -310 (U-tailing). RNA was incubated at 75°C and ramped down to 50°C before SuperScript III was added for reverse transcription. PCR amplification using Ex Taq DNA polymerase Hot Start (Clontech) or Q5 Hot Start High-Fidelity 2x Master Mix was run using the AUAP primer combined with TS-O-00974 for EPgV-1 and with RU-O-20147 or RU-O-20148 for EPgV-2. For EPgV-2, a nested or semi-nested PCR was run using primer RU-O-20147 or RU-O-20148 combined with the corresponding cDNA primer. Cycling parameters using Ex Taq were 94°C for 2 min, followed by 40 cycles of 94°C for 30 s, 55°C for 30 s and 72°C for 30 s, and using Q5 they were 98°C for 30 s, followed by 40 cycles of 98°C for 10 s, 52°C for 10 s and 72°C for 1 min with a final extension at 72°C for 5 min.

The presence of EPgV-1 and EPgV-2 negative strand RNA in bone marrow, liver, and spleen biopsies of infected horses was investigated by 3’ end tailing of negative strand (-)RNA. The 3’ end of viral RNA was tailed with homopolymers of ATP or GTP using Yeast Poly(A) Polymerase (USB Affymetrix), and cDNA was synthesized using SuperScript III with primers TS-O-00178 and TS-O-00525, respectively. RNA was incubated at 65°C for 5 min, the temperature was ramped down to 48°C and enzyme was added. 1^st^ round PCR using Q5 Hot Start High-Fidelity 2x Master Mix (NEB) was run using primers TS-O-00653 (EPgV-1) or TS-O-00131 (EPgV-2) combined with AUAP. The 2^nd^ round PCR was run using primer TS-O-00654 (EPgV-1) or TS-O-00312 (EPgV-2) combined with AUAP. Cycling parameters were 98°C for 1 min, followed by 40 cycles of 98°C for 30 s, 52°C for 40 s and 72°C for 1 min plus a final extension at 72°C for 5 min. This procedure further confirmed the sequence of the positive strand (+)RNA 5’ end.

For complete ORF amplification of EPgV-1 and -2 RNA, total RNA was extracted from 250 μL horse serum using TRIzol LS Reagent (Thermo Fisher Scientific). After addition of chloroform and centrifugation, the aqueous phase was mixed with 450 μL anhydrous ethanol and transferred to an RNA Clean & Concentrator-5 column (Zymo Research) for downstream RNA purification and concentration. RT was performed with Maxima H Minus Reverse Transcriptase (Thermo Fisher Scientific). Samples were pre-incubated in the absence of the RT enzyme at 65°C for 2 min prior to the reaction. RT was carried out at 50°C for 2 h in the presence of RNase inhibitors (Promega) using 0.1 μM primers EPgV-RT-Full and TDAV-Full-RT. Amplification of the complete ORF was performed using Q5 High-Fidelity Hot start DNA Polymerase (NEB), including high GC Enhancer, and the primer pairs TS-O-01215 and TS-O-01218 (EPgV-1) or TDAV-Full-F and TDAV-Full-R (EPgV-2), at 0.5 μM each. PCR cycling parameters were 98°C for 30 s, followed by 35 cycles of 98°C for 10 s, 65°C for 10 s and 72°C for 9.5 min, with a final extension at 72°C for 10 min. Amplified DNA was purified using DNA Clean & Concentrator columns (Zymo Research), and libraries for deep sequencing were prepared using the NEBNext Ultra II FS DNA Library Prep Kit for Illumina (NEB). Quality of DNA libraries was validated using a 2100 Bioanalyzer Instrument (Agilent). The Qubit dsDNA High-Sensitivity Assay Kit (Thermo Fisher Scientific) was used to quantify DNA libraries in order to pool these in equimolar concentrations prior to denaturation. Pooled libraries were loaded on a MiSeq v3 150 cycle flow-cell, and sequencing performed on a MiSeq benchtop sequencer (Illumina) at the Department of Clinical Microbiology (Hvidovre Hospital, Copenhagen).

### Engineering of EPgV full-length clones

To obtain the consensus sequence of the EPgV-1 C35 isolate, RT was done using SuperScript III and random nonamers. PCRs of overlapping fragments were done using KOD Hot Start DNA Polymerase (Novagen) and primers as described in S2 Table. This yielded 5–8 clones each, of six overlapping fragments (I-VI), from which the consensus sequence was determined. A full-length consensus clone, pC35 was assembled in the pGEM9zf backbone using standard PCR and restriction digest molecular biology from overlapping clones of fragments I-VI, and a 3’ UTR clone [12]. A *BsrGI* site was engineered immediately downstream of the 3’ UTR for linearization. A replication-deficient clone, pC35-GNN, was constructed by mutating the NS5B active site, GDD, by site-directed mutagenesis.

For complete ORF sequencing of EPgV-2 isolates, including TDAV, RNA was reverse transcribed using SuperScript III (Thermo Fisher) and primers TS-O-00173 or -530. Overlapping PCR amplicons were generated using Q5 polymerase with GC-enhancer (NEB) and primers (I) TS-O-00133 and -157, (II) TS-O-00135 and -165, (III) TS-O-00527 and -528, or (IV) TS-O-00151 and -531. Sanger sequencing of these showed no differences from the published deep sequencing data [13]. A full-length consensus clone, pTDAV, was synthesized (GenScript) based on the published sequence with the 5’ and 3’ ends, as determined above. This was transferred to the pUC19 backbone. A *BsrGI* site was engineered immediately downstream of the 3’ UTR for linearization. A comparison of the final clones, consensus sequence, and published sequences are shown in S3 and S4 Tables.

### Cell culture and preparation of EFLCs

Huh-7.5 cells were maintained in DMEM supplemented with 10% FBS; E. Derm (NBL-6) and BHK-J in MEM with 10% FBS; MDBK in DMEM with 10% BVDV-free FBS, non-essential amino acids and 0.1mM sodium pyruvate; and Vero in serum-free Opti-Pro with 4 mM glutamine. Vero cells were split using TrypLE Express (Life Technologies), and other cell lines with regular trypsin.

To prepare EFLCs, an equine fetal liver (80 days of gestation) was placed in RPMI-1640 on ice, and EFLCs were isolated the same day, as previously described [57]. 3.2x10^4^ cells in W10 Plating Medium were plated per well of a 96-well plate. After attachment, EFLCs were cultivated in serum-free Hepatocyte Defined Medium (HDM; BD Biosciences).

### *In vitro* transcription, transfection and electroporation

RNA was *in vitro* transcribed from *BsrGI*-linearized DNA template plasmids using T7 RiboMAX Express Large Scale RNA Production System (Promega). RNA was treated with RQ1 DNase on ice for 30 min and purified on RNeasy columns (Qiagen), including an additional on-column DNase I digestion. For transfection into cell lines, 2.5 μg RNA was mixed with 5 μL Lipofectamine 2000 in 500 μL OptiMEM, incubated 20 min and added to 4x10^5^ cells in 2 mL media in 6-well plates. Cells were split every 2–3 days, and supernatant and cell pellets were stored at -80°C for subsequent analysis by RT-qPCR.

### *In vivo* RNA inoculation and monitoring of infection

For laparoscopic intrasplenic inoculation with TDAV RNA, procaine penicillin, gentamicin and flunixin meglumine were administered just prior to the procedure, the horse was sedated with detomidine and morphine, and the site was blocked with mepivicaine. A laparoscopic cannula (Stryker Corp.) was inserted via an incision through the skin, a video camera was attached to allow visualization of the spleen, and the abdomen was insufflated to 15 mmHg, using CO_2_ gas. An 18-gauge needle was inserted and video guided to the spleen. Five batches of *in vitro* transcribed TDAV RNA, a total of ~400 μg RNA, were then injected into five different sites of the spleen. For subsequent intrasplenic or intra bone marrow RNA inoculations with C35 EPgV-1 RNA, horses were sedated with xylazine and the site was blocked with 2% lidocaine. An 18-gauge needle was ultrasound guided into the spleen, or a 14-gauge Jamshidi bone marrow biopsy needle was inserted into a *sternebra*. Five batches of *in vitro* transcribed RNA, on average a total of ~400 μg RNA, were then injected into five different sites of the spleen or one site in the bone marrow.

Samples were processed on automated analyzers ADVIA 2120i (Siemens Healthcare Diagnostics) for hematology and Cobas C501 (Roche Diagnostics) for clinical biochemical profiles, weekly prior to and following the procedure for 28 weeks. The inflammatory markers serum amyloid A (SAA) and iron indices were monitored weekly for 8 weeks. Hemogram parameters and reference intervals were: hematocrit, 34–46%; hemoglobin, 11.8–15.9 g/dL; red blood cell count, 6.6–9.7 million/μL; mean corpuscular volume, 43–55 fL; mean corpuscular hemoglobin, 15–20 pg; mean corpuscular hemoglobin concentration 34–37 g/dL; red cell distribution width, 16.3–19.3%; nucleated red blood cells, 0/100 white blood cells; white blood cells, 5.2–10.1 thousand/μL; segmented neutrophils, 2.7–6.6 thousand/μL; band neutrophils 0.0–0.1 thousand/μL; lymphocytes, 1.2–4.9 thousand/μL; monocytes, 0.0–0.6 thousand/μL; eosinophils, 0.0–1.2 thousand/μL; basophils, 0.0–0.2 thousand/μL; platelet count, 94–232 thousand/μL; mean platelet volume, 5.3–8.4 fL; total protein, 5.2–7.8 g/dL. Blood smears were examined manually to confirm automated results. Biochemical markers examined were: aspartate aminotransferase (AST), sorbitol dehydrogenase (SDH), glutamate dehydrogenase (GLDH), gamma glutamyltransferase (GGT), bile acids, total, direct, and indirect bilirubin, creatine kinase (CK), triglycerides, SAA, serum iron, total iron binding capacity (TIBC), and ferritin (FE) saturation. Reference intervals: AST, 222–489 U/L; SDH, 1–6 U/L; GLDH, 2–10 U/L; GGT, 8–33 U/L; Bile acids, 2–10 μmol/L; Total bilirubin, 0.5–2.1 mg/dL; Direct bilirubin, 0.1–0.3 mg/dL; Indirect bilirubin, 0.3–2.0 mg/dL; Creatine kinase, 171–567 U/L; Triglycerides, 14–65 mg/dL; SAA, 0–8 μg/mL; serum iron, 95–217 μg/dL; TIBC. 289–535 μg/dL; and FE saturation, 27–56%. Sample preparation and RNA analysis from serum and biopsies were as described above.

TDAV reactive antibodies were measured using the LIPS assay as described elsewhere [58], here using partial TDAV NS3 (translated nt 3936–4745 of the TDAV clone) as antigen.

PBMCs were prepared by Ficoll density gradient centrifugation and cell populations were measured with flow cytometry using 2 panels (**Table 1**). All wash steps were 2 mL PBS, and all labeling was performed at 4°C. For panel M, cells were blocked with 2% FBS for 15 min and then incubated with unconjugated antibody (anti-CD16) for 30 min. Cells were washed, blocked with 10% goat serum for 15 min, and incubated with secondary antibody for 30 min. Cells were incubated with a cocktail of the remaining conjugated monoclonal antibodies to surface antigens for 30 min and washed. Streptavidin-pacific orange was applied for 30 min to label biotinylated antibodies, cells were washed and resuspended in PBS with 7AAD viability stain. For panel T, a fixable viability marker was first applied for 30 min, washed, and the surface cocktail followed by streptavidin was applied as for Panel M. Cells were fixed (eBioscience Intracellular fixation and permeabilization buffer set, Thermo Fisher Scientific) at room temperature for 30 min, washed in permeabilization buffer, incubated with the intracellular marker anti-Ki67 for 30 min, washed and resuspended in PBS. Fluorescence was measured on a Gallios flow cytometer (Beckman Coulter) with a minimum 100,000 events collected. Analysis was performed with FlowJo version 10.6.1 (FlowJo LLC). Single color controls were used to set the compensation matrix. Gating strategies for flow panel M and T are shown in **S5 and S6 Figs**, respectively.

**Table 1:** Antibodies used in flow cytometric phenotyping of PBMC.

Target antigen	Panel used in	Ab source	Target species	Clone	Conjugate
Pan-Ig	M	BioRad MCA1899PE	Equine	CVS36	RPE
CD3	M, T	UC Davis Dr. Stott	Equine	UC-F6G	AF647
CD4	T	BioRad MCA1078F	Equine	CVS4	FITC
CD8	T	BioRad MCA2385PE	Equine	CVS8	RPE
Ki67	T	BD Biosciences 661283	Human	B56	PE-Cy7
CD21	T	BD Biosciences 562966	Human	B-ly4	BV421
CD16	M	Antczak lab	Equine	1A2.D11	None
CD14	M, T	Wagner lab	Equine	105	biotin
Viability		BioLegend 420404	n/a	7AAD	n/a
Viability		ThermoFisher L10119	n/a	Live/Dead Near IR	n/a

### *In situ* hybridization

Formalin-fixed, paraffin-embedded (FFPE) bone marrow core biopsies were labeled with RNAScope 2.5 HD-Red ISH (Advanced Cell Diagnostics). Two twenty ZZ probe pairs, V-Pegivirus-D-NS3 (catalog no. 830241), targeting base pairs 3936–4863 of the NS3 gene of EPgV-2 (NC_038433.1), and V-Pegivirus-E-NS3 (catalog no. 829961), targeting base pairs 3541–5367 of the NS3 gene of EPgV-1 (NC_020902.1), were designed. ISH was performed with a 15 min antigen retrieval step. Positive (Ec-PPIB) and negative (dap-B) control probes were used (ACD catalog numbers 462351 and 310043, respectively). Probes were applied to EPgV-2 and EPgV-1 RT-PCR positive bone marrow.

### Immunohistochemistry

Formalin-fixed, paraffin-embedded (FFPE) bone marrow core biopsies were labeled with mouse anti-double strand RNA (J2, Scions) at 1:100 dilution. A polyclonal goat anti-mouse IgG (H+L) Horseradish peroxidase conjugated antibody (Jackson ImmunoResearch Laboratories, Catalog #115-035-062) was used as secondary antibody. The AEC substrate kit (Abcam) was used for development. Probes were applied to EPgV-2 and EPgV-1 RT-PCR positive bone marrow. Pre-infection bone marrow and secondary antibody only controls were included.

## Data availability

The complete C35 and TDAV genome sequences, the near-complete IA1, IA2 and NV1 sequences, as well as partial NS3 sequences of isolates shown in Fig 1 were deposited to GenBank under accession no. MT276199-MT276223. Flow cytometry data were deposited to Flow Repository with ID: FR-FCM-Z2KY.

## Supporting information

S1 Fig *In situ* hybridization (ISH) for EPgV-1 and immunohistochemistry (IHC) for double stranded RNA. **(A)** ISH performed on bone marrow core biopsies collected at week 28 post inoculation (Horse X, upper panel, Fig 4A). Shown is a positive control probe (PPIB; pink hybridization), C35 probe (pink hybridization) and negative control probe (DapB). The long arrow indicates a single pink C35 probe hybridization spot. Arrowheads indicate reddish-brown background pigment deposits, likely hemosiderin, which in some instances are difficult to distinguish from positive signal. C35 samples were decalcified, while TDAV samples were not. It is unlikely, however, that the ambiguous results for C35 samples are explained by this, given that the PPIB positive control gave expected results on decalcified samples. **(B)** IHC using J2 anti-dsRNA antibody with horseradish-peroxidase conjugated secondary antibody developed with a red chromogen. The J2 antibody was applied to equine parvovirus-hepatitis (EqPV-H) infected liver as a positive control, to TDAV PCR-positive bone marrow (Horse J), to C35 PCR-positive bone marrow (Horse X), and to pre-infection bone marrow (Horse X) as negative biological control. Secondary antibody only was applied as technical negative control (Horse X). Arrows indicate positive label, arrowheads indicate non-specific background.
(TIF)

S2 Fig Structure prediction of 5’ UTRs. The structures of the C35 **(A)** and TDAV **(B)** 5’ UTRs were predicted using MFOLD [57] and guided by covariant base-pairs from alignment of isolates. Predicted structures of 3’ terminal regions are inserted in boxes. Only little direct similarity is evident between 5’ UTR structures of EPgV-1 and -2; this is indicated in green. Other features are colored as indicated.
(TIF)

S3 Fig Alignment of EPgV 3’ UTR sequences. Variable nucleotide positions are typed in red. Poly(C) regions are shaded in blue. For EPgV-1, repeat sequence elements (RSE) are shaded in orange (type 1) and yellow (type 2). Stem-loops consistent with folding prediction (MFOLD) of all isolates in several of the energetically most favourable predictions are indicated with two arrows directed toward each other pointing to the loop. For EPgV-1, the stems of RSE loops may vary in lengths depending on the isolate and are indicated based on isolate C35.
(TIF)

S4 Fig Transfection of C35 consensus RNA into various cell lines. RNA transcripts from pC35 and pC35-GNN were transfected into the indicated cell lines. Replication was assessed by RT-qPCR on intracellular RNA over time.
(TIF)

S5 Fig Gating strategy for flow panel M. **(A)** Events were gated first to exclude dead cells (7AAD vs FS-A; 7AAD^neg^) and then **(B)** to exclude doublets (FS-H vs. FS-A). **(C)** Cells were then separated by CD3 and CD14 expression. **(D)** T-cells were identified as CD3^pos^CD14^neg^CD16^neg^, while NK-like cells were classified as CD3^pos^CD14^neg^CD16^pos^. **(E)** B-cells were identified as CD3^neg^CD14^neg^PanIg^pos^. **(F)** Monocytes were identified as CD3^neg^CD14^pos^ and were sub-typed as classical (CD16^low^) or alternatively activated (CD16^hi^).
(PDF)

S6 Fig Gating strategy for flow panel T. **(A)** Events were gated first to exclude dead cells (Live/Dead Near IR vs FS-A; LD^neg^) and then **(B)** to exclude doublets (FS-H vs. FS-W). **(C)** CD21^pos^ B-cells were excluded. **(D)** CD14^pos^ monocytes were excluded. **(E)** CD3^pos^ cells were included. **(F)** Cells were gated on CD4 and CD8. **(G)** Ki67 expression in CD4^pos^CD8^neg^ cells. **(H)** Ki67 expression in CD4^pos^CD8^pos^ cells. **(I)** Ki67 expression in CD4^neg^CD8^neg^ cells. **(J)** Ki67 expression in CD4^neg^CD8^pos^ cells.
(TIF)

S1 Table Primer sequences. (PDF)

S2 Table C35 consensus determination. (PDF)

S3 Table Comparison of C35 consensus clone sequence. (PDF)

S4 Table Comparison of TDAV consensus clone sequence. (PDF)

S1 Data Mutation frequency tables for C35 and TDAV. Related to Fig 4B. Provided as an Excel file. Shown are the genomic nucleotide position (POS), reference nucleotide (REF), mutation nucleotide (ALT), frequency in percentage for individual samples (sample names refer to Fig 4B), type of mutation (Functional_Class), codon change and amino acid change.
(XLSX)
