# Supplementary figures and images for: Equine pegiviruses cause persistent infection of bone marrow and are not associated with hepatitis
Source: PLoS Pathog. 2020 Jul 10;16(7):e1008677. doi: 10.1371/journal.ppat.1008677 (PMC7375656; doi:10.1371/journal.ppat.1008677)

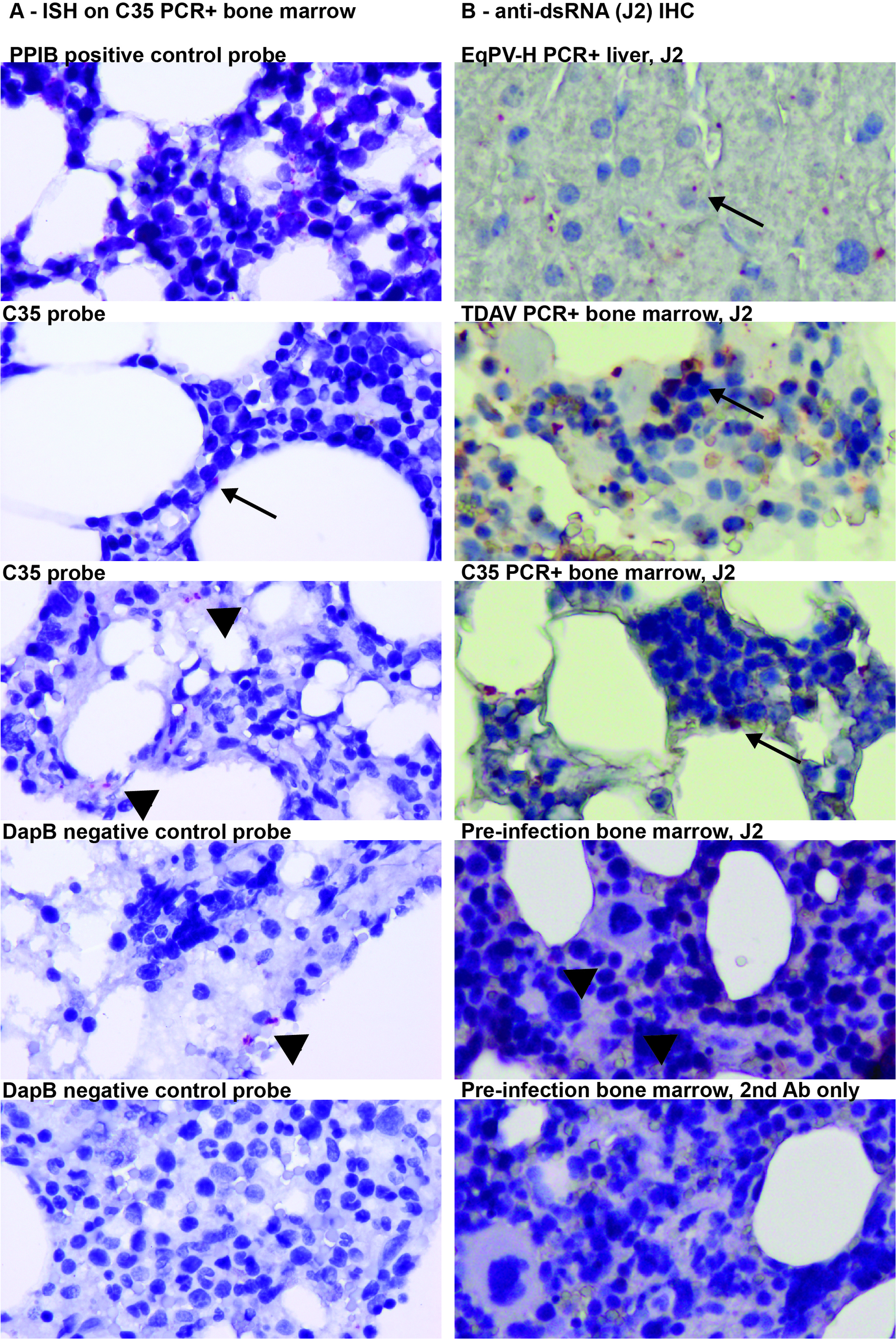

Supplement: S1 Fig — (A) ISH performed on bone marrow core biopsies collected at week 28 post inoculation (Horse X, upper panel, Fig 4A). Shown is a positive control probe (PPIB; pink hybridization), C35 probe (pink hybridization) and negative control probe (DapB). The long arrow indicates a single pink C35 probe hybridization spot. Arrowheads indicate reddish-brown background pigment deposits, likely hemosiderin, which in some instances are difficult to distinguish from positive signal. C35 samples were decalcified, while TDAV samples were not. It is unlikely, however, that the ambiguous results for C35 samples are explained by this, given that the PPIB positive control gave expected results on decalcified samples. (B) IHC using J2 anti-dsRNA antibody with horseradish-peroxidase conjugated secondary antibody developed with a red chromogen. The J2 antibody was applied to equine parvovirus-hepatitis (EqPV-H) infected liver as a positive control, to TDAV PCR-positive bone marrow (Horse J), to C35 PCR-positive bone marrow (Horse X), and to pre-infection bone marrow (Horse X) as negative biological control. Secondary antibody only was applied as technical negative control (Horse X). Arrows indicate positive label, arrowheads indicate non-specific background. (TIF) [file ppat.1008677.s001.tif]

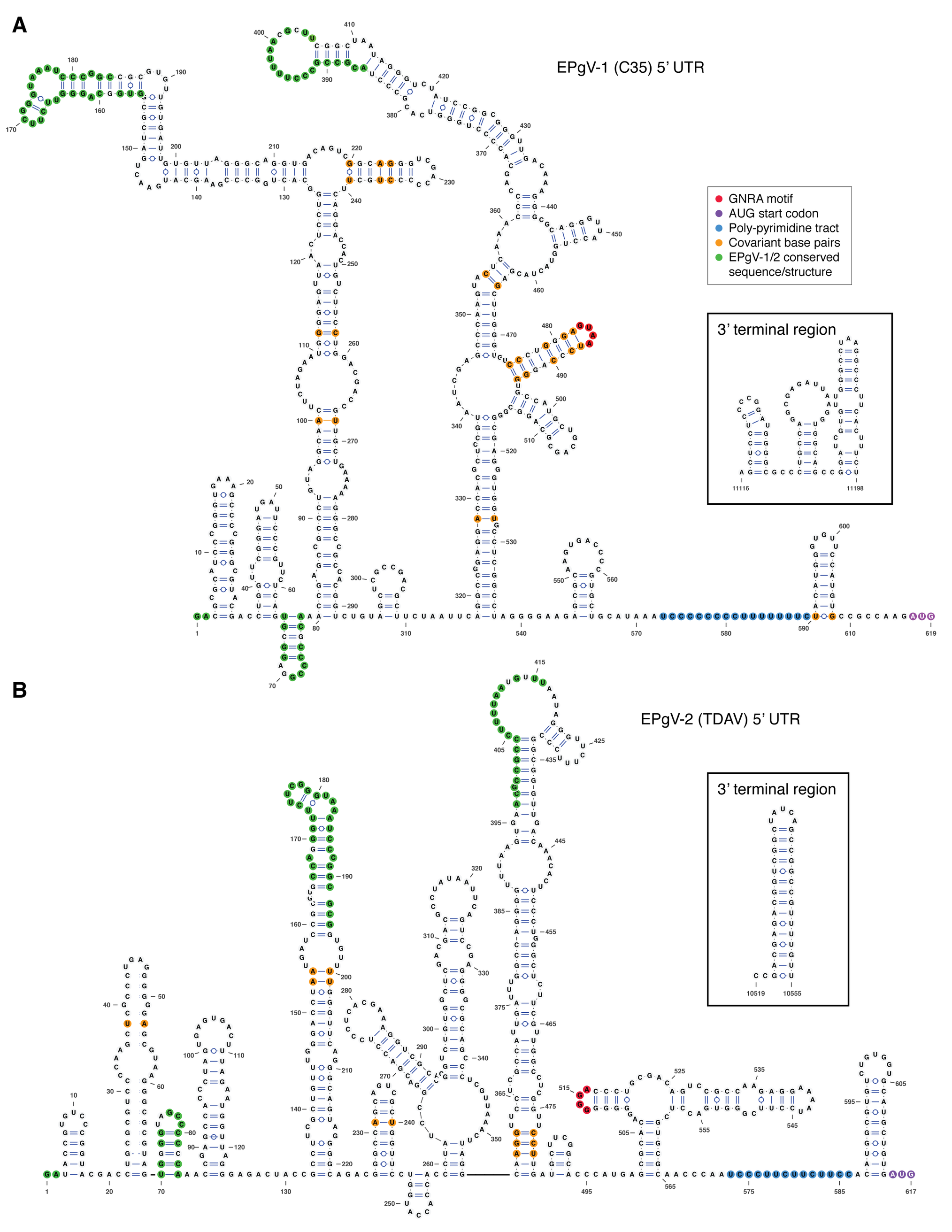

Supplement: S2 Fig — The structures of the C35 (A) and TDAV (B) 5’ UTRs were predicted using MFOLD [57] and guided by covariant base-pairs from alignment of isolates. Predicted structures of 3’ terminal regions are inserted in boxes. Only little direct similarity is evident between 5’ UTR structures of EPgV-1 and -2; this is indicated in green. Other features are colored as indicated. (TIF) [file ppat.1008677.s002.tif]

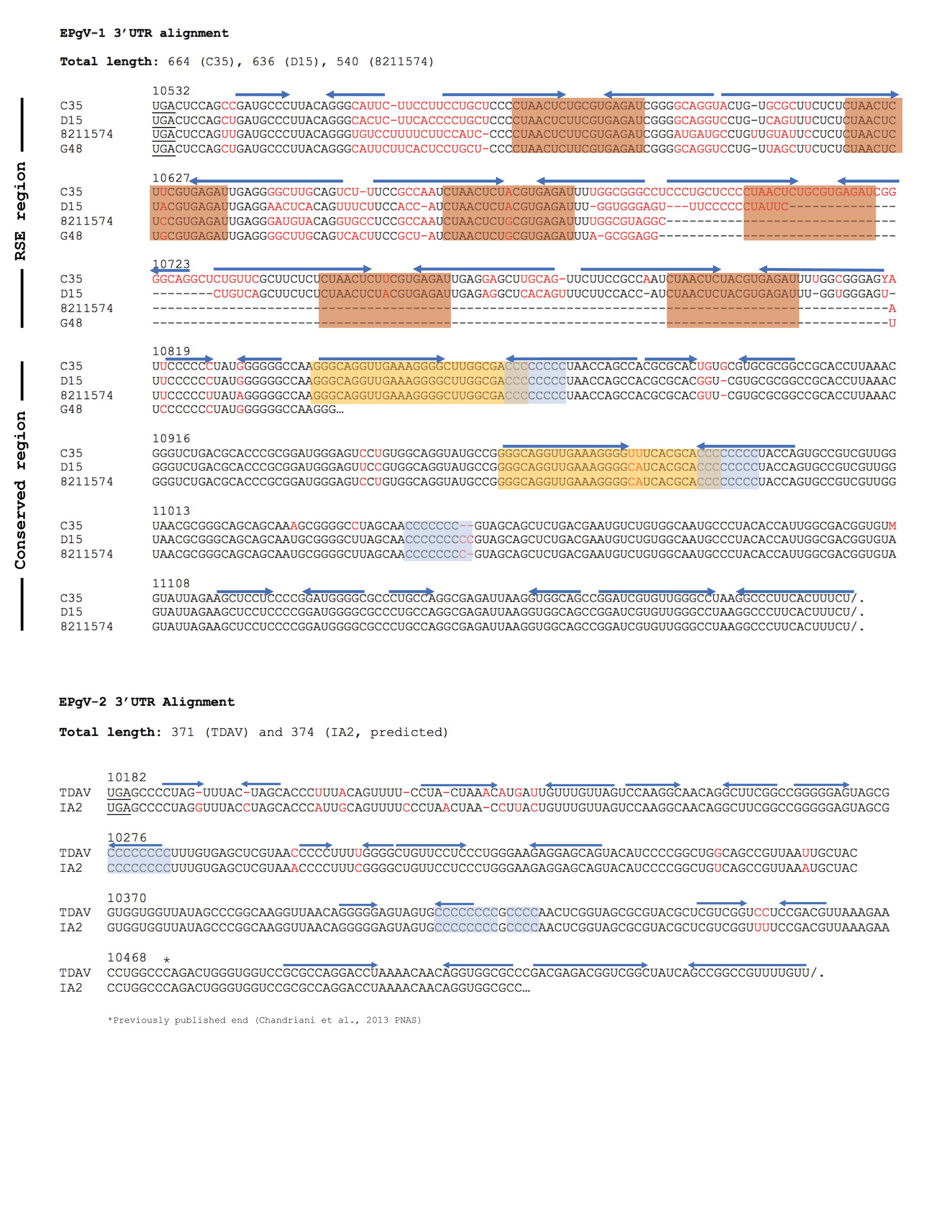

Supplement: S3 Fig — Variable nucleotide positions are typed in red. Poly(C) regions are shaded in blue. For EPgV-1, repeat sequence elements (RSE) are shaded in orange (type 1) and yellow (type 2). Stem-loops consistent with folding prediction (MFOLD) of all isolates in several of the energetically most favourable predictions are indicated with two arrows directed toward each other pointing to the loop. For EPgV-1, the stems of RSE loops may vary in lengths depending on the isolate and are indicated based on isolate C35. (TIF) [file ppat.1008677.s003.tif]

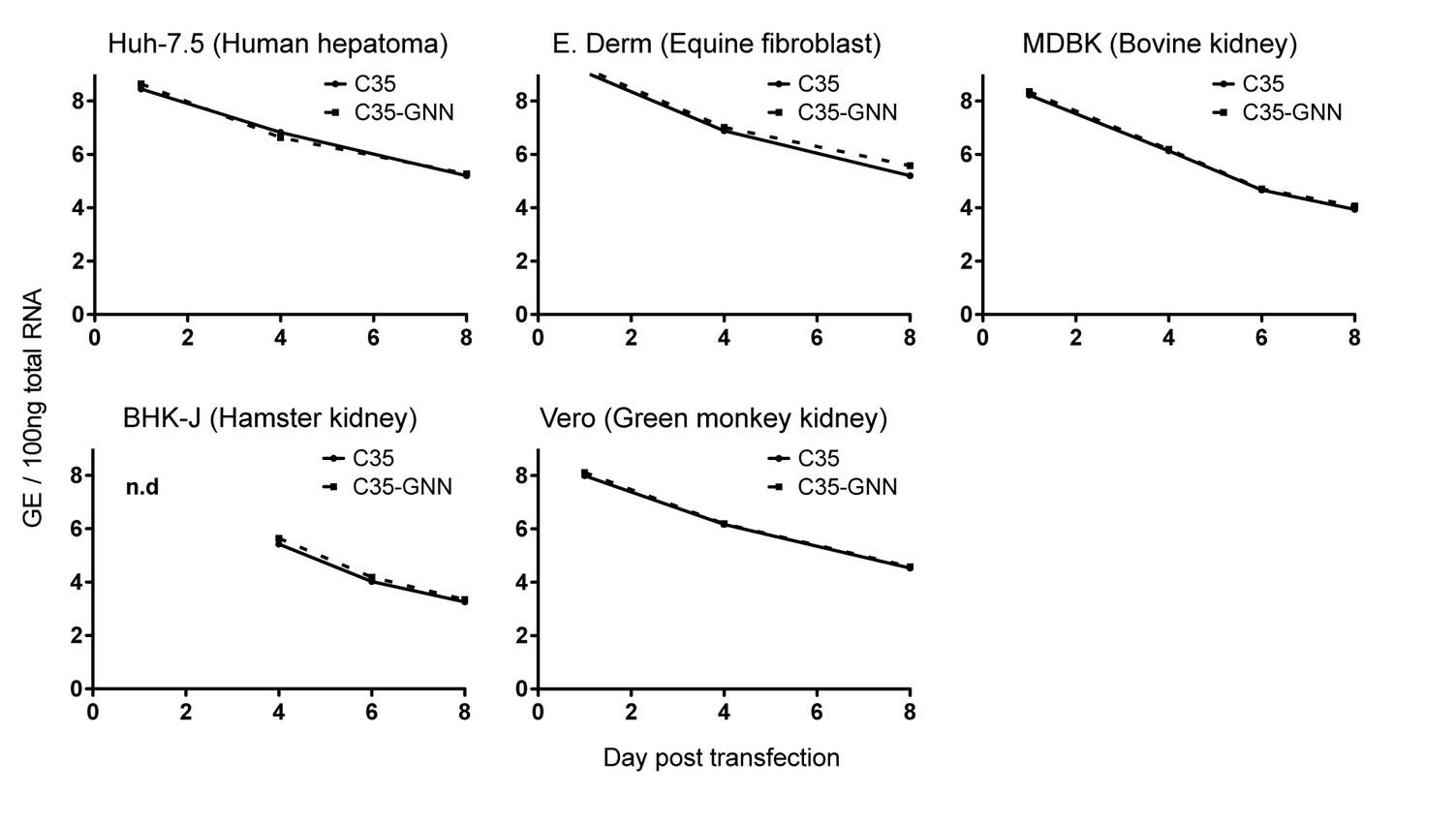

Supplement: S4 Fig — RNA transcripts from pC35 and pC35-GNN were transfected into the indicated cell lines. Replication was assessed by RT-qPCR on intracellular RNA over time. (TIF) [file ppat.1008677.s004.tif]

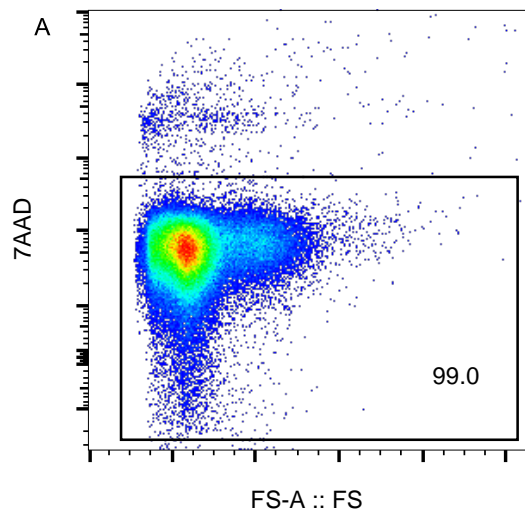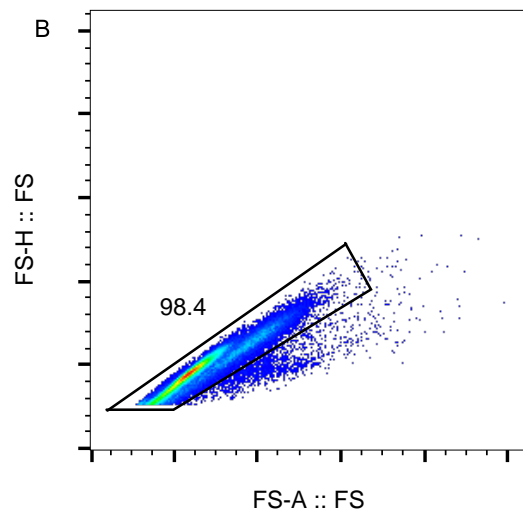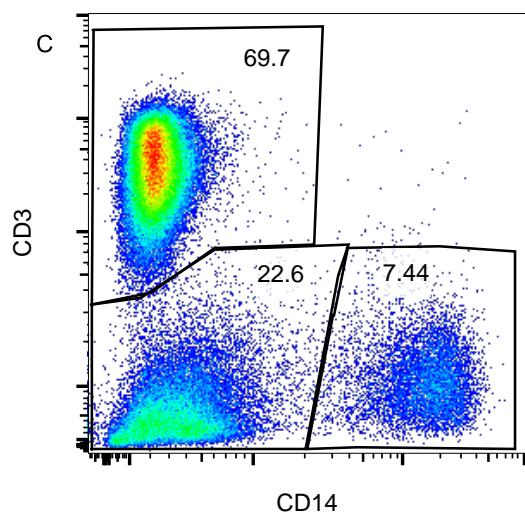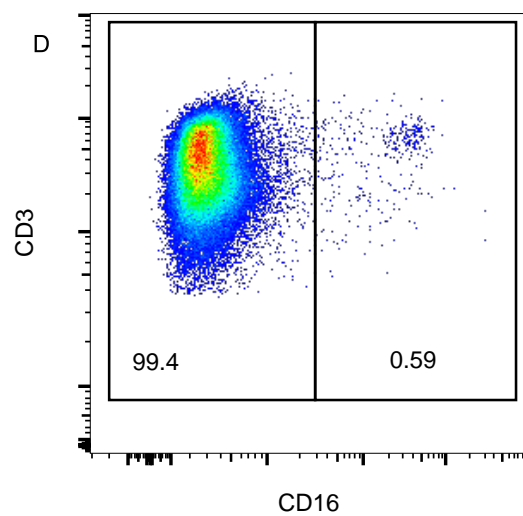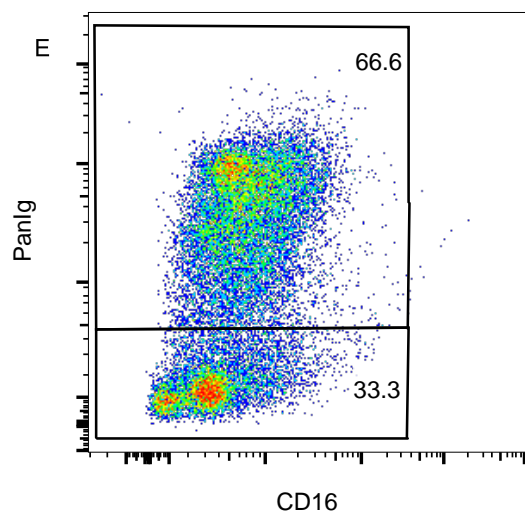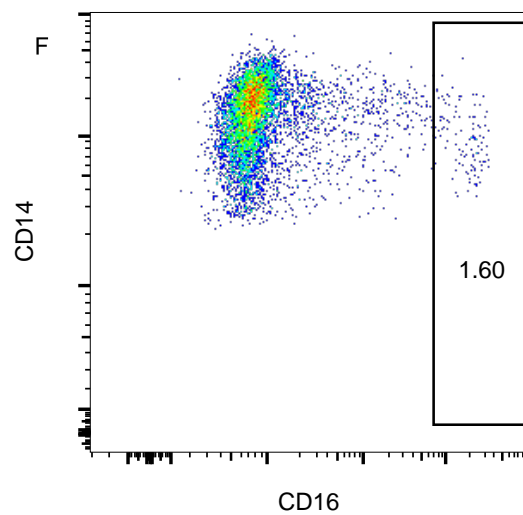

Supplement: S5 Fig — (A) Events were gated first to exclude dead cells (7AAD vs FS-A; 7AADneg) and then (B) to exclude doublets (FS-H vs. FS-A). (C) Cells were then separated by CD3 and CD14 expression. (D) T-cells were identified as CD3posCD14negCD16neg, while NK-like cells were classified as CD3posCD14negCD16pos. (E) B-cells were identified as CD3negCD14negPanIgpos. (F) Monocytes were identified as CD3negCD14pos and were sub-typed as classical (CD16low) or alternatively activated (CD16hi). (PDF) [file ppat.1008677.s005.pdf]

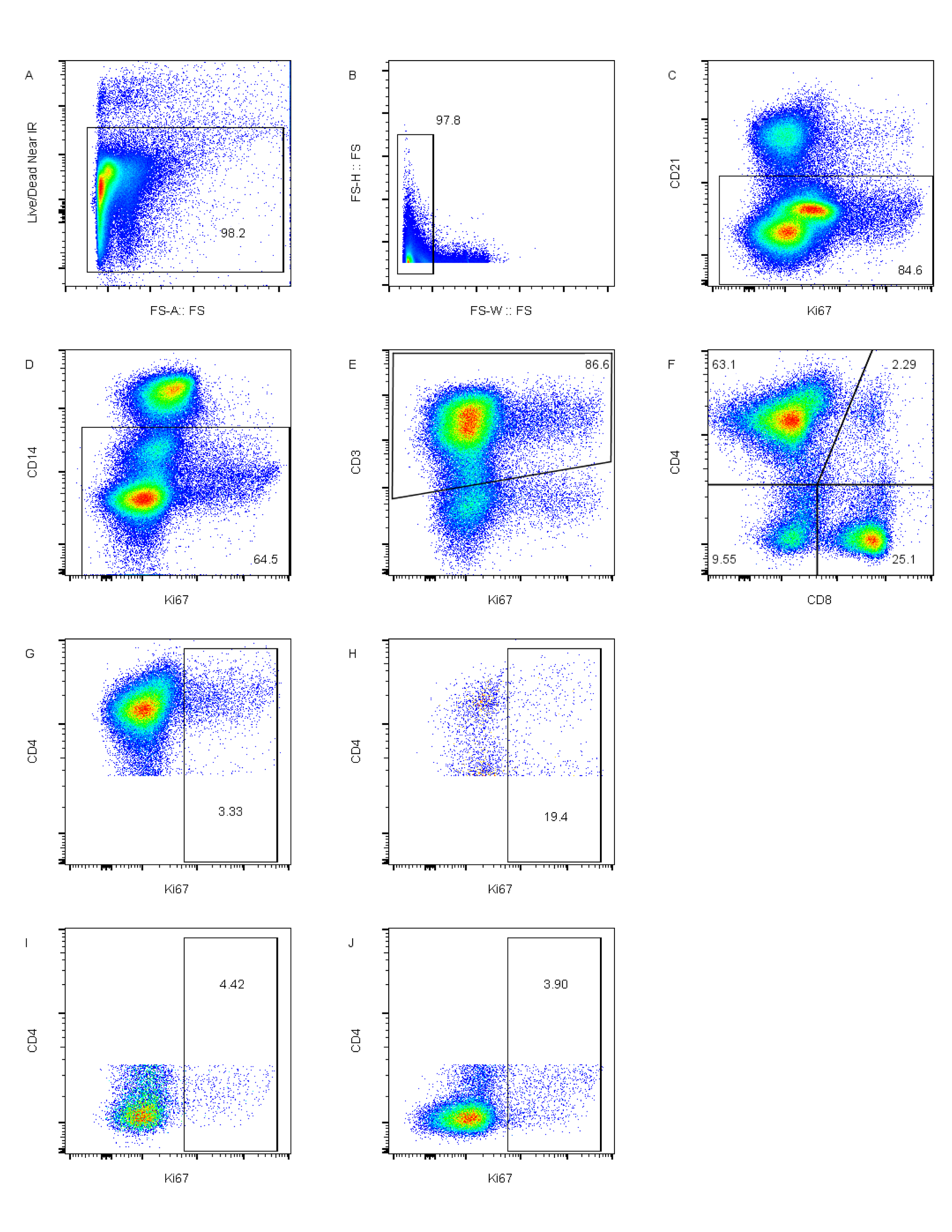

Supplement: S6 Fig — (A) Events were gated first to exclude dead cells (Live/Dead Near IR vs FS-A; LDneg) and then (B) to exclude doublets (FS-H vs. FS-W). (C) CD21pos B-cells were excluded. (D) CD14pos monocytes were excluded. (E) CD3pos cells were included. (F) Cells were gated on CD4 and CD8. (G) Ki67 expression in CD4posCD8neg cells. (H) Ki67 expression in CD4posCD8pos cells. (I) Ki67 expression in CD4negCD8neg cells. (J) Ki67 expression in CD4negCD8pos cells. (TIF) [file ppat.1008677.s006.tif]
