# Supplementary material for: Equine pegiviruses cause persistent infection of bone marrow and are not associated with hepatitis
Source: PLoS Pathog. 2020 Jul 10;16(7):e1008677. doi: 10.1371/journal.ppat.1008677 (PMC7375656; doi:10.1371/journal.ppat.1008677)
Supplement: S1 Table — (PDF) [file ppat.1008677.s007.pdf]

Tomlinson, Wolfisberg et al.: Equine pegiviruses cause persistent infection of bone marrow and are not associated with hepatitis

S1 Table. Primer sequences.

Name	Sequence
AAP	GGCCACGCGTCGACTAGTACGGGIIGGGIIGGGIIG
AUAP	GGCCACGCGTCGACTAGTAC
RU-O-18176	CCGCTGGAAGTGACTGACACCCCCCCC
RU-O-18594	TGGTCAGCGGCCGCTAGCTAATACGACTCACTATAGACGCGCATCCCGGGTGAAAGCCC
RU-O-18596	GACGCGCATCCCGGGTGAAAGC
RU-O-18597	TGTTTGGGCTTACCCTCTTG
RU-O-18598	TTGGGCTTACCCTCTTGATG
RU-O-18599	GCATGAGGAAGTGGAGGTTG
RU-O-18600	TACTACGCTCTTGCTGCTTCC
RU-O-18601	TATGGCTATCGAGCGTGATG
RU-O-18603	AAGTTGGGTAGGGGAAGAGG
RU-O-18604	CAAAGCAAAGTTGGGTAGGG
RU-O-18605	TGACAAGTATACGCGCAACC
RU-O-18606	TGGCATCCTCAATGTGTGTAG
RU-O-18608	ACGAGCAGCTACCTGAGTCC
RU-O-18681	ACCTCCACGGAAAGTCACTC
RU-O-18682	AACAGAACCTCCACGGAAAG
RU-O-18684	TTCCCCCATTGGAATTTAGAG
RU-O-18686	ACGGTAATGGCCATAGGAAG
RU-O-18692	CTCGGCCTCTTGATGATGAC
RU-O-18695	TGTCTGCACTGAGGACCATC
RU-O-18868	CGTCAGACCCGTTTAAGGTG
RU-O-18869	GTGAAACCCCTTTCAACCTG
RU-O-19037	GGGACICAGAGCACICCNCA
RU-O-19038	TCGGTGCTIACCACCACIAGRTC
RU-O-20147	GGCTACTGGCTTCTGTTCTG
RU-O-20148	TTGTTAGTCCAAGGCAACAG
TS-O-00130	CCCAAACCGAGCCRCCCT
TS-O-00131	CGGACTGAATTATAGGCGTCG

TS-O-00132	/56-FAM/CCGGGATTTACCCGAAGAACCCTG/3IABkFQ/
TS-O-00133	TGATACCGTGTCCCGGTACGACCTCG
TS-O-00135	TATCTGTCAAACTGCTGTGATC
TS-O-00151	GCTTGGTAGCCAAGGCCGTCCTGGGTGATGC
TS-O-00157	TGAGGCCAAGCAAGCCGACAG
TS-O-00165	TGCACTCTGACCGTTGGACTC
TS-O-00173	TTGCCAATCTGGAGCTCAAGG
TS-O-00178	GGCCACGCGTCGACTAGTACTTTTTTTTTTTTTTTTTTTTVN
TS-O-00310	GGCCACGCGTCGACTAGTACAAAAAAAAAAAAAAAAAAAABN
TS-O-00311	CGCCGGGAAAGAACCCTATTAAAC
TS-O-00312	GATTTACCCGAAGAACCCTGG
TS-O-00373	ACGCAGAGCAAGATTACCTATGC
TS-O-00374	CCTGGTGGAGTAGCAGTAGC
TS-O-00375	/FAM/ACGCTGACGTCGTGATTTGCGACGA/BHQ_1/
TS-O-00525	GGCCACGCGTCGACTAGTACCCCCCCCCDN
TS-O-00527	GGACAGCGTGCCCAGGGATGC
TS-O-00528	CCTGAGAGCTATAGTCCTCTTCCG
TS-O-00530	TCCTCTTCCCAGGGAGGAACAG
TS-O-00531	AAAGGGGGTTACGAGCTCACAA
TS-O-00653	GACTGTCACCTGCCCTAACACAATC
TS-O-00654	GAACCCTGCCACCGCGATCAGT
TS-O-00974	GTGGATGGCCTATTTCCTTG
TS-O-01214	GTCGGCGAGCTACAGA
TS-O-01215	CACTGGCCCGAAGCATGAAC
TS-O-01218	GGCTGCCACCTTAATCTCGC
EPgV-RT-Full	AGAAAGTGAAGGGCCTTAG
TDAV-Full-F	GATACCGTGTCCCGGTACGACCTGCGCGTCCCC
TDAV-Full-R	GGCCAGGTTCTTTAACGTCGGAGGA
TDAV-Full-RT	GGCCAGGTTCTTTAACGTC
TDAV-236F	CCGTGTACCCAATGCCTGTAG
TDAV-692R	GTGGCAACAGACGGGTTCA
