# Supplementary material for: Equine pegiviruses cause persistent infection of bone marrow and are not associated with hepatitis
Source: PLoS Pathog. 2020 Jul 10;16(7):e1008677. doi: 10.1371/journal.ppat.1008677 (PMC7375656; doi:10.1371/journal.ppat.1008677)
Supplement: S2 Table — (PDF) [file ppat.1008677.s008.pdf]

Tomlinson, Wolfisberg et al.: Equine pegiviruses cause persistent infection of bone marrow and are not associated with hepatitis

S2 Table. C35 consensus determination.

Fragment	Start	End	Forward	Reverse	Forward (nested)	Reverse (nested)
I	1	2302	RU-O-18596	RU-O-18604	RU-O-18594	RU-O-18603
II	2107	5127	RU-O-18597	RU-O-18606	RU-O-18598	RU-O-18605
III	4976	7090	RU-O-18599	RU-O-18682	RU-O-18600	RU-O-18681
IV	5622	8215	RU-O-18692	RU-O-18608	-	-
V	7870	9378	RU-O-18601	RU-O-18686	-	-
VI	9210	10925	RU-O-18695	RU-O-18869	RU-O-18684	RU-O-18868
