# Supplementary material for: Equine pegiviruses cause persistent infection of bone marrow and are not associated with hepatitis
Source: PLoS Pathog. 2020 Jul 10;16(7):e1008677. doi: 10.1371/journal.ppat.1008677 (PMC7375656; doi:10.1371/journal.ppat.1008677)
Supplement: S3 Table — (PDF) [file ppat.1008677.s009.pdf]

Tomlinson, Wolfisberg et al.: Equine pegiviruses cause persistent infection of bone marrow and are not associated with hepatitis

S3 Table. Comparison of C35 consensus clone sequence.

**Position**	**AA change**	**KC410872**	**Consensus (# clones)**	**pC35 clone**
587	UTR	T	TT (4/7)	TT
798	nc	C	T	T
801	nc	T	C	C
2523	nc	T	C	C
2697	nc	C	T	T
2712	nc	T	C	C
3777	nc	T	C	C
3969	nc	C	T	T
4648	nc	C	T	T
4677	nc	A	G	G
4968	nc	C	T	T
6696	nc	C	T	T
7950	nc	A	G	G
9231	nc	C	T	T
9312	nc	A	G	G
9314	Q → R	A	G	G
9792	nc	G	A	A
9925	nc	T	C	C
10062	nc	A	G	G
10586	UTR	C	T	T
