# Supplementary material for: Equine pegiviruses cause persistent infection of bone marrow and are not associated with hepatitis
Source: PLoS Pathog. 2020 Jul 10;16(7):e1008677. doi: 10.1371/journal.ppat.1008677 (PMC7375656; doi:10.1371/journal.ppat.1008677)
Supplement: S4 Table — (PDF) [file ppat.1008677.s010.pdf]

Tomlinson, Wolfisberg et al.: Equine pegiviruses cause persistent infection of bone marrow and are not associated with hepatitis

S4 Table. Comparison of TDAV consensus clone sequence.

Position (pTDAV numbering)	AA change	KC145265	Consensus	pTDAV clone
1	UTR	TG	G	G
23	UTR	TC	T	T
146	UTR	GGGG	GGG	GGG
